# Prefrontal electrophysiological biomarkers and mechanism-based drug effects in a rat model of alcohol addiction

**DOI:** 10.1038/s41398-024-03189-z

**Published:** 2024-12-05

**Authors:** Bettina Habelt, Dzmitry Afanasenkau, Cindy Schwarz, Kevin Domanegg, Martin Kuchar, Carsten Werner, Ivan R. Minev, Rainer Spanagel, Marcus W. Meinhardt, Nadine Bernhardt

**Affiliations:** 1 Department of Psychiatry and Psychotherapy, Faculty of Medicine Carl Gustav Carus, Technische Universität Dresden, Dresden, Germany; 2 Leibniz Institute of Polymer Research Dresden, Dresden, Germany; 3 Biotechnology Center (BIOTEC), Center for Molecular and Cellular Bioengineering (CMCB), Technische Universität Dresden, Dresden, Germany; 4 Institute of Psychopharmacology, Central Institute of Mental Health, Medical Faculty Mannheim, University of Heidelberg, Mannheim, Germany; 5 Forensic Laboratory of Biologically Active Substances, Department of Chemistry of Natural Compounds, University of Chemistry and Technology Prague, Prague, Czech Republic; 6 Psychedelic Research Center, National Institute of Mental Health, Klecany, Czech Republic; 7 Else Kröner Fresenius Center for Digital Health, Faculty of Medicine Carl Gustav Carus, Technische Universität Dresden, Dresden, Germany; 8 German Center for Mental Health (DZPG), partner site Mannheim/Heidelberg/Ulm, Mannheim, Germany; 9 Department of Molecular Neuroimaging, Central Institute of Mental Health, Medical Faculty Mannheim, University of Heidelberg, Heidelberg, Germany

**Keywords:** Biomarkers, Neuroscience

## Abstract

Patients with alcohol use disorder (AUD) who seek treatment show highly variable outcomes. A precision medicine approach with biomarkers responsive to new treatments is warranted to overcome this limitation. Promising biomarkers relate to prefrontal control mechanisms that are severely disturbed in AUD. This results in reduced inhibitory control of compulsive behavior and, eventually, relapse. We reasoned here that prefrontal dysfunction, which underlies vulnerability to relapse, is evidenced by altered neuroelectric signatures and should be restored by pharmacological interventions that specifically target prefrontal dysfunction. To test this, we applied our recently developed biocompatible neuroprosthesis to measure prefrontal neural function in a well-established rat model of alcohol addiction and relapse. We monitored neural oscillations and event-related potentials in awake alcohol-dependent rats during abstinence and following treatment with psilocybin or LY379268, agonists of the serotonin 2A receptor (5-HT_2A_R), and the metabotropic glutamate receptor 2 (mGluR2), that are known to reduce prefrontal dysfunction and relapse. Electrophysiological impairments in alcohol-dependent rats are reduced amplitudes of P1N1 and N1P2 components and attenuated event-related oscillatory activity. Psilocybin and LY379268 were able to restore these impairments. Furthermore, alcohol-dependent animals displayed a dominance in higher beta frequencies indicative of a state of hyperarousal that is prone to relapse, which particularly psilocybin was able to counteract. In summary, we provide prefrontal markers indicative of relapse and treatment response, especially for psychedelic drugs.

## Introduction

Substance use disorders are a severe health issue worldwide. With more than three million deaths each year, the highest impact is attributed to the abuse of alcohol [1, 2]. Treatment options for alcohol use disorder (AUD) include pharmacological interventions (e.g. acamprosate and naltrexone) and psychotherapy (e.g. cognitive behavioral therapy). However, patients with AUD who seek treatment show highly variable outcomes, limiting the acceptance of currently available interventions in clinical practice [3, 4]. A wealth of theoretical and empirical evidence thus strongly supports efforts towards a precision medicine approach with adequate biomarkers to identify and target pathophysiological mechanisms that will likely respond best to a given treatment [5, 6]. Potential biomarkers of clinical state and treatment responsiveness include electrophysiological brain activity measures of neural oscillations and event-related potentials (ERPs) that represent the neural basis of sensory information processing and higher-order cognitive abilities such as attention, working memory, decision-making, and behavioral control – prefrontal functions that are strongly disturbed in AUD [7, 8].

Resting-state neural oscillations describe a continuous, rhythmic, and repetitive electrical activity generated spontaneously by temporally and spatially synchronized neurons and reflect a rather fundamental brain state [9, 10]. Brain oscillations are subdivided into different frequency bands, most commonly delta (1–4 Hz), theta (4–8 Hz), alpha (8–12 Hz), beta (12–30 Hz), and gamma (>30 Hz) [11]. Chronic alcohol consumption increases oscillatory power within all frequency bands, but most prominently in the beta band [12–16]. Higher-frequency beta activity has been associated with a state of hyperarousal and increased relapse probability [12].

In contrast, event-related oscillations (ERO) refer to time-frequency measurements in response to motor, sensory, or cognitive events [17]. When tested for executive functioning during Go/NoGo tasks, AUD patients display reduced ERO power in the delta, theta, and alpha range in response to NoGo stimuli [18, 19], indicating impaired behavioral inhibition and cognitive control. Disturbed impulse control has also been related to beta oscillations, which are dominant in sensorimotor functioning and display a reduced power in binge drinkers during NoGo task conditions [20]. Superposition and phase synchronization of EROs further determine the formation of ERP components [17, 21]. These stimulus-evoked voltage deflections are time-locked local positive or negative maxima within a continuously recorded electroencephalogram. They are commonly named according to their polarity (P = positive, N = negative) and time (in ms post-stimulus) or order of appearance [22].

Alcohol consumption in humans [23, 24] and ethanol exposure in animal models [25–28] reduce amplitudes and/or increase latencies of multiple ERP components, pointing to alterations of the entire information processing system. Increased salience of alcohol-related cues in combination with a lack of inhibitory resources are the central neurocognitive mechanisms underlying relapse, with the P3 being the most reliable ERP component to predict relapse risk and treatment success [29–31].

It is recognized that the described cognitive impairments and limited behavioral control observed in AUD relate to disturbances primarily within prefrontocortical networks [32, 33]. Thus, the present study builds on the assumption that (i) aberrant neuroelectric signatures in AUD represent impaired prefrontal function that underlies vulnerability to relapse, and (ii) pharmacological interventions that address such a vulnerable state can restore altered electrophysiological activity.

We previously developed an electrocorticographic (ECoG) interface [26] tailored to capture prefrontal neural activity, and we here applied this new technology to a well-established animal model for alcohol addiction and relapse behavior. In this model, rats are subjected to long-term voluntary alcohol consumption in a four-bottle procedure, repeatedly interrupted with abstinence periods. The re-presentation of alcohol following deprivation induces relapse-like drinking - a temporary increase in alcohol intake over baseline drinking referred to as the alcohol-deprivation effect (ADE) [34]. The development of compulsive drinking behavior is further characterized by insensitivity to taste adulteration with quinine, a loss of circadian drinking patterns, and a shift towards drinking highly concentrated alcohol solutions to rapidly increase blood alcohol concentrations and achieve intoxication during a relapse situation. In addition, alcohol-dependent rats that derive from this model show tolerance and physical as well as anxiety-related withdrawal symptoms [35–37]. The ADE model has been utilized in various preclinical and translational alcohol studies and has helped identify new treatment targets with good predictive validity [35, 38].

After assessing prefrontal electrophysiological signatures in the ADE rat model, we tested two drug treatments that we expected to interfere with prefrontal dysfunction and relapse behavior. One of the therapeutic targets is the metabotropic glutamate receptor 2 (mGluR2), a key regulator of glutamate release. Prefrontal mGluR2 expression and function are strongly diminished in animal models of alcohol addiction and severe AUD cases [39, 40]. Consequently, it has been proposed to use mGluR2 agonists such as LY379268 to counteract this diminished prefrontal function, providing preclinical evidence of its potential to attenuate alcohol-seeking and relapse-like behavior [40–44]. Recently, we demonstrated that virally restoring normal prefrontal mGluR2 levels can prevent cognitive impairment and craving in alcohol-dependent rats and that the normalization of mGluR2 is also possible via a single administration of the psychedelic agent psilocybin which ultimately reduced relapse-like behavior [40]. Psilocybin seems promising in treating AUD since a recent clinical trial using psilocybin in combination with psychotherapy demonstrated a significant reduction of heavy drinking days in AUD patients [45]. The behavioral and therapeutic effects of psilocybin are primarily attributed to the activation of the serotonin 2A receptor (5-HT_2A_R) [46] and its modulation by other pathways, including the physical interaction with the mGluR2 [39]. Both mGluR2 and 5-HT_2A_R are enriched in prefrontal areas. This supports our hypothesis that their activation can restore altered ERPs and neural oscillations in alcohol-dependent rats, thereby improving cognitive functioning and reducing the risk of relapse.

## Materials and Methods

### Ethics approval

Animals used and all applied investigations have been approved by the institutional ethics commissions of TU Dresden and the Central Institute of Mental Health, Mannheim, and the regional authorities of the federated states of Saxony (Landesdirektion Sachsen) and Baden-Württemberg (Regierungspräsidium Karlsruhe). Experiments were performed in accordance with the guidelines of Directive 2010/63/EU on the protection of animals used for scientific purposes of the European Commission with great attention to avoid suffering and to reduce the number of animals used [47].

### Animals

We used male Wistar rats from the breeding colony at the CIMH. Rats were housed in single cages (Makrolon®, Type III, Tecniplast Deutschland GmbH) on sawdust bedding (Ssniff - Bedding 3/4 S, Altrogge) with Bed-r’Nest material (Datesand Ltd.). Pelleted food (V1534-300, ssniff Spezialdiäten GmbH) and water were available ad libitum. Housing rooms were temperature (20 - 22°C) and humidity (40 - 55%) controlled with a 12 h automatic light-dark cycle (lights on at 6.00 am).

### Long-term alcohol consumption with repeated deprivation periods

Following two weeks of habituation to the animal room, rats (*n* = 10) were given ad libitum access to ethanol (VWR International GmbH) solutions of 5%, 10%, and 20% (v/v) besides tap water. Special bottle caps that minimize spillage and evaporation and allow constant ethanol concentrations were used [48] and the positions of bottles were changed weekly. Concurrent access to several alcohol concentrations has been shown to increase the magnitude and duration of the ADE [34, 35]. After eight weeks of continuous alcohol availability, bottles with alcohol solution were removed from the cages and reintroduced after a deprivation period of two weeks. Phases of free access to alcohol and deprivation subsequently alternated randomly with variable durations of drinking between 4 – 6 weeks and deprivation lasting 2–3 weeks, aiming to prevent habituation and behavioral adjustment.

**Fig. 1:**
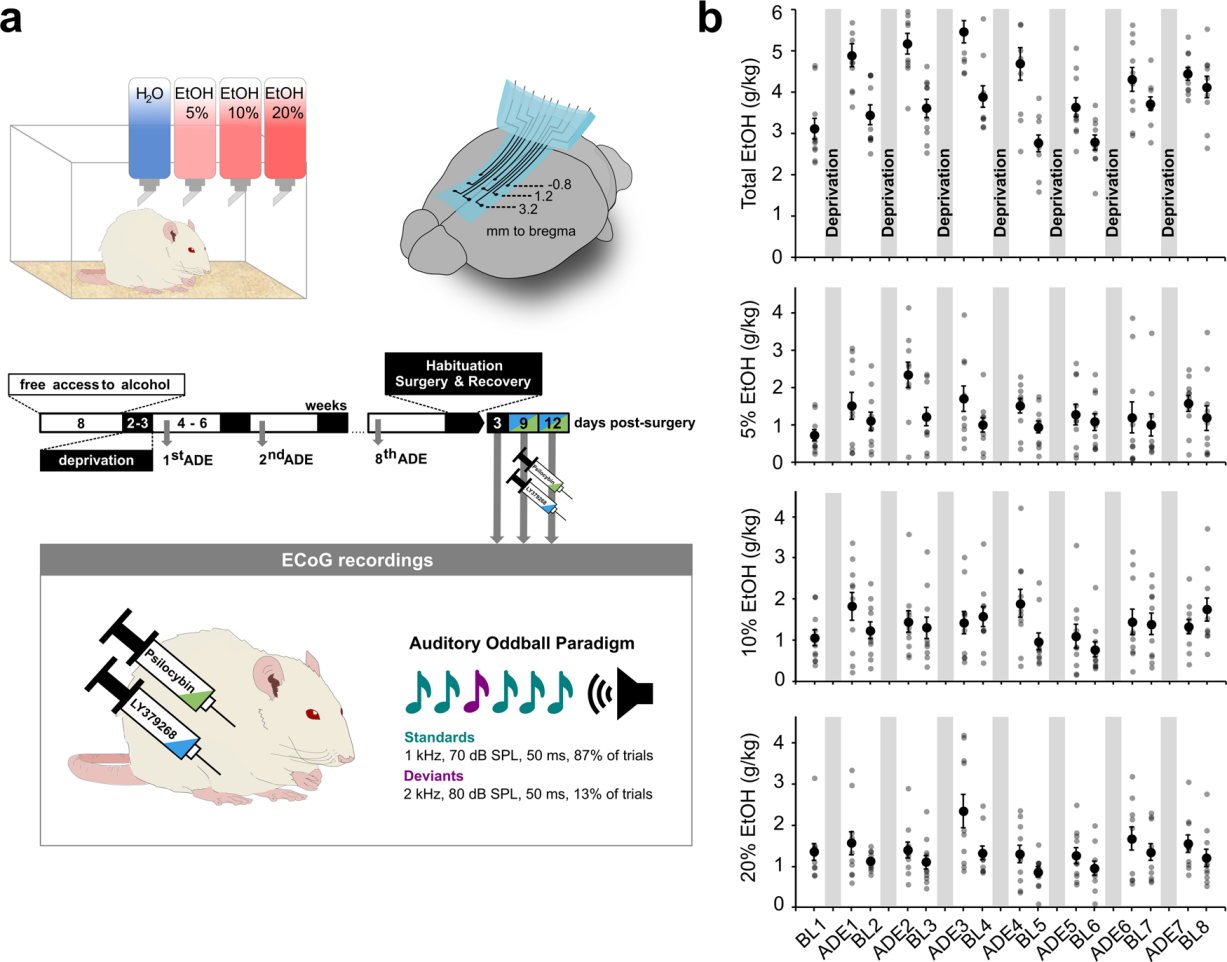
Experimental timeline of long-term alcohol consumption and ECoG recordings. **a** In alternating cycles, animals had access to either alcohol solutions of 5%, 10%, and 20% ethanol besides water (free access to alcohol) or to water alone (deprivation). Alcohol consumption phases were initially 8 weeks and then between 4 – 6 weeks, interspersed with deprivation periods of 2 – 3 weeks. Following the final (8^th^) alcohol-drinking cycle, rats were habituated to the recording set-up and underwent stereotactic surgery to implant the neuroprosthetic interface. Alcohol-detoxified animals underwent electrocorticographic (ECoG) recordings during a two-tone auditory oddball paradigm initially three days post-surgery without any further interventions. Subsequent recording sessions were performed 30 min following i.p. administration of psilocybin or LY379268 in a randomized order nine- and twelve-days post-surgery, respectively. The auditory oddball paradigm consisted of altogether 1620 tones (1344 standards, 276 deviants), delivered in 6 × 5 min blocks with varying breaks (<1 min) between blocks. **b** Alcohol consumption patterns throughout the experimental timeline. Data are presented as individual data points (grey) as well as daily means ± standard error of the mean of pure EtOH in g per kg over all animals (black) during the last week of a drinking phase (baselines, BL) and on the first day following periods of abstinence (i.e. Alcohol Deprivation Effect (ADE)).

The long-term alcohol exposure procedure, including all drinking and deprivation phases, continued over a year. Consumption of each of the three alcohol solutions was followed for each rat along the entire length of the protocol. During the final two-week period of alcohol deprivation, the animals were habituated to the recording set-up and underwent surgery to implant the neuroprosthetic device (Fig. 1a). Ten alcohol-naïve control animals underwent the same procedure [26]. All rats were in an advanced adult age >PND180, with animals in the control group ranging between 6-12 months and the experimental group being 14 months at the recording time.

### Manufacturing and implantation of neuroprosthetic interfaces

The procedures to fabricate, characterize, and implant the used neuroprosthetics have been described in detail before [26, 49, 50]. Briefly, micro-EcoG interfaces were produced by an additive manufacturing approach using the 3D bioprinter 3DDiscovery^TM^ Evolution (regenHU Ltd.). The devices consisted of soft silicone for the base (DOWSIL™ SE 1700, Dow Inc., Midland, USA) and isolating (SE 734, Dow Inc., Midland, USA) layers embedding a 3 × 3 electrode array of conductive platinum ink (chemPUR). The electrode interconnects were attached to stainless steel microwires (∅: 0.23 mm, 7SS-2T, Science Products GmbH) soldiered to a plug-in connector (BKL 10120653, BKL-Electronic Kreimendahl GmbH). An additional microwire was fixed to a microscrew drilled into the skull during surgery and served as a reference electrode. Implantation was performed under subcutaneous (s.c.) anesthesia (fentanyl (0.005 mg/kg, Hameln Pharma), midazolam (2 mg/kg, Ratiopharm), medetomidine hydrochloride (0.135 mg/kg, Orion Pharma) in a stereotactic surgery involving trepanation of the skull (∅ 6.0 mm, 330205486001060, Meisinger) to position the devices epidurally on the prefrontal cortex with the frontal electrode row located at 3.2 mm anterior to bregma. External parts of the implant were fixed to the skull using dental cement (Paladur, Kulzer GmbH), and the wound was sutured. After completion of the surgery, anesthesia was antagonized (s.c., naloxone hydrochloride (0.12 mg/kg, Inresa Arzneimittel GmbH), flumazenil (0.2 mg/kg), atipamezolhydrochloride (0.75 mg/kg, Orion Pharma). Animals received meloxicam (1 mg/kg, s.c., Boehringer Ingelheim Vetmedica GmbH) as an analgesic right after surgery and the following day.

### Electrocorticography recording and acute pharmacological modulation of neural activity

ECoG recordings without any further interventions were implemented three days after the animals had undergone surgery. One animal at a time was placed in a rodent sling (Lomir Biomedical Inc.) to reduce movement artefacts within an electrically shielded and sound-insulated audiometry booth. Recordings were performed at a sampling rate of 3 kHz using the Intan RHD2000 USB interface system cable connected to the implant plug-in module. The initial recording measured a state of abstinence in alcohol-dependent rats compared to matched alcohol-naïve rats.

To elicit ERPs an auditory oddball paradigm was used, which is a classical method for studying prefrontal function and higher-order cognitive processes [51]. Here, a subject’s ability to detect and respond to infrequent “oddball” tones among more frequent standard sounds relies on intact prefrontal circuitries that guide selective attention and executive control. Auditory ERPs have translational value as they show similar characteristics and responses to changes in stimuli (e.g. loudness, frequency, probability) in both humans and animals [52, 53] and have been indexed as biomarkers of pathophysiological mechanisms [29]. Sound stimuli were presented through a stereo loudspeaker located at a distance of 40 cm and an angle of 45° centrally above the animal’s head. Auditory stimuli have been generated using the Psychophysics Toolbox (Version 3) for Matlab (Version R2019b, The Mathworks Inc.) and were composed of frequent (standards: 50 ms, 1 kHz, 70 dB sound pressure level (SPL), 87% of trials) and rare (deviants: 50 ms, 2 kHz, 80 dB SPL, 13% of trials) sinusoidal sounds with 5-ms onset/offset ramps, presented in 6 blocks of 5 min with 1 s interstimulus interval. Deviant sounds have been interspersed with at least one standard tone to avoid successive occurrences.

On days 6 and 9 after initial recordings, alcohol-dependent rats randomly received intraperitoneal (i.p.) injections of psilocybin (2.5 mg/kg, 3-[2-(dimethylamino) ethyl]-1H-indol-4-yl] dihydrogen phosphate (purity 99.7%) obtained from the University of Chemistry and Technology Prague, Czech Republic, dissolved in Ampuwa (Braun Melsungen AG) or LY379268 (1 mg/kg, 1 *R*,4 *R*,5*S*,6 *R*)-4-Amino-2-oxabicyclo[3.1.0]hexane-4,6-dicarboxylic acid, Tocris Bioscience) each 30 min before recording. The acute treatment regime and respective dosing were chosen based on previous data that demonstrated the strongest effect size in reducing alcohol relapse without adverse effects for psilocybin (*d* = 1.17 for the 2.5 mg/kg dose as opposed to d = 1.00 for the 1 mg/kg dose) and LY379268 (*d* = 0.85 for 1 mg/kg, only dose without side effects) [40, 44, 54].

To capture drug-induced changes in resting-state neural activity, we performed continuous 5-minute recordings right before administering psilocybin or LY379268, and directly after the auditory oddball paradigm described above. Pre- and post-application recordings were approximately one hour apart.

#### Event-related potentials and oscillatory activity

Automated data processing was performed using the EEGLAB toolbox [55] (Version 2019.1) for Matlab. Following offline filtering using a 0.1–45 Hz bandpass finite impulse response filter (Kaiser windowed, Kaiser β = 5.65, filter length 54,330 points), data were segmented in epochs between -100 and 700 ms relative to stimulus onset separately for standard and deviant sounds and baseline-corrected using the pre-stimulus interval between -100 ms to 0 ms. Artefacts and noisy channels were identified and excluded based on a delta criterion of 500 µV. Automated output was quality checked by visual inspection before averaging epochs for single subjects and over all animals (grand average). As neural responses to the frequent standard sound rarely displayed pronounced amplitudes, indicating habituation effects due to the high repetition rate [56], subsequent analysis was performed on the difference curves (deviant-minus-standard responses). ERP peak latencies were attributed to predefined time intervals and confirmed by visual inspection: P1: 20–70 ms, N1: 35–120 ms, P2: 60–260 ms, N2: 100–320 ms, P3: 130–600 ms. The amplitudes of the ERP components were calculated as peak-to-peak amplitudes (P1N1, N1P2, P2N2, N2P3).

Oscillatory power in the delta (1–4 Hz), theta (4–8 Hz), alpha (8–12 Hz), beta (12–30 Hz), and gamma (30–45 Hz) bands was determined by applying the function pop_newtimef.m in EEGLAB based on a Fast Fourier transform using 400 datapoints and a pad ratio of 64. The resulting event-related spectral perturbation (ERSP) was calculated as decibels (dB) (≙ 10*log_10_ (µV^2^/Hz)). For each frequency band and the entire frequency range, we additionally calculated the maximum ERSP and the frequency value and latency of the maximum ERSP.

#### Resting-state neural oscillations

Data were initially bandpass filtered, as described above and segmented into 2 s-epochs with 50% overlap. Following the exclusion of bad epochs (δ = 500 µV, confirmed by visual inspection), we applied the EEGLAB function pop_spectopo.m to determine channel spectra based on the Welch method. The resulting power spectral density (PSD) in the 1–45 Hz frequency range was calculated as dB.

### Statistics

Statistical analyses were conducted using SPSS® (Version 28, IBM Corp.) and R (Version 4.2.3, R Foundation for Statistical Computing). Differences in ERPs (peak latencies, amplitudes) and EROs (bandpower (ERSP), latency and frequency of max. power within each frequency band and over the whole frequency range) induced by psilocybin and LY379268 were examined by applying within-subjects repeated measures analysis of variance (rmANOVA) with factors treatment and channel. Multiple comparisons were adjusted using the Sidak correction. Likewise, rmANOVA was applied to analyze resting-state oscillatory activity (PSD) before vs. after administration of psilocybin and LY379268.

Neural parameters of alcohol- and drug-naïve controls were compared with those of long-term alcohol consumers and each pharmacological intervention by applying a between-subjects ANOVA with factors treatment and channel and Sidak adjustment for multiple comparisons.

To explore the potential impact of individual alcohol consumption patterns and susceptibility to the ADE on designated neural parameters, we performed Spearman correlation analyses using the averaged amounts of alcohol (g/kg body weight) consumed by each rat per day during the last week of a drinking phase (=baseline consumption, BL) and on the first day following periods of abstinence (=Alcohol Deprivation Effect (ADE)) as well as relapse intensities (difference of ADE and previous BL). *Partial* Spearman correlation was performed to relate alcohol consumption patterns with the neural activity following pharmacotreatments, controlling for neural activity without drug administration.

Results were interpreted based on significant *p*-values (*p* < 0.05) in addition to effect sizes, as the latter better reflect the impact of treatment independent of sample size. Given the ongoing criticism of p-values [57], we support the use of multiple statistical parameters to interpret results.

## Results

### Impact of long-term alcohol consumption on neural activity

Alcohol consumption patterns throughout the experimental timeline are illustrated in Fig. 1b and S1. Mean baseline total alcohol intake (g/kg/day ± standard deviation (SD)) was 3.43 ± 0.54, with rats consuming 1.02 ± 0.54 of 5%, 1.25 ± 0.45 of 10% and 1.16 ± 0.40 of the 20% alcohol solution, respectively. Following deprivation, animals displayed increased consumption of all alcohol concentrations compared to baseline drinking (5%: 1.58 ± 0.60, *p* = 0.006; 10%: 1.49 ± 0.47, *p* = 0.11; 20%: 1.59 ± 0.58, *p* = 0.034, Total: 4.66 ± 0.64, *p* = < 0.001, two-sided paired t-tests) revealing a pronounced ADE.

**Fig. 2:**
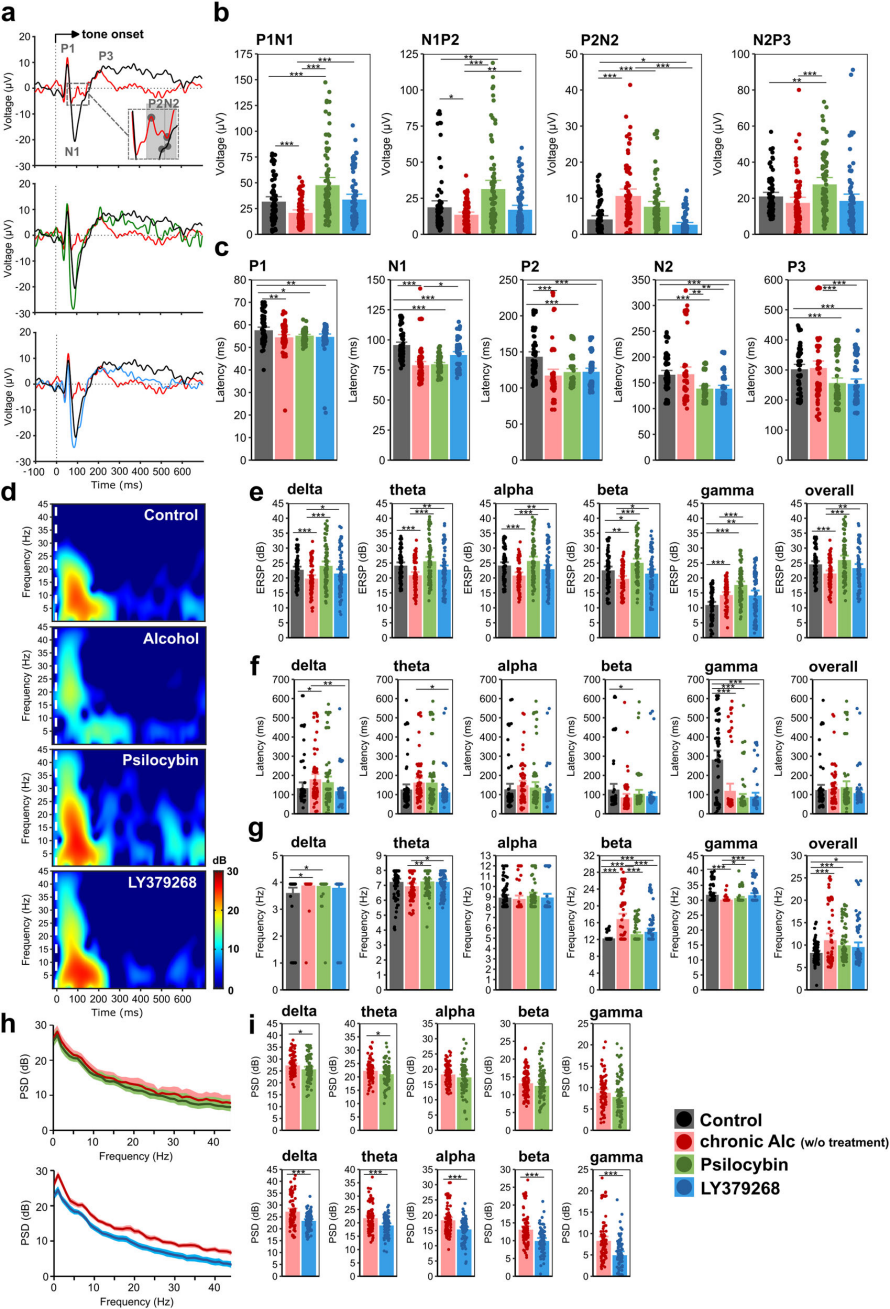
Impact of chronic alcohol consumption and administration of psilocybin and LY379268 on prefrontal electrophysiological activity. **a** Grand average deviant-minus-standard auditory event-related potential (ERP) difference curves at the frontocentral (FC) electrode. **b** Peak-to-peak amplitudes of P1N1, N1P2, P2N2 and N2P3 components. **c** ERP peak latencies of P1, N1, P2, N2 and P3 components. **d** Grand average deviant-minus-standard event-related oscillatory (ERO) activity at the FC channel. Data is given as event-related spectral perturbation (ERSP) in decibels (dB). **e** Maximum ERO activity (as ERSP in dB) within delta (1-4 Hz), theta (4-8 Hz), alpha (8-12 Hz), beta (12-30 Hz), and gamma ( > 30 Hz) frequency bands and over the whole frequency range. **f** Latencies of maximum ERO activity within the delta, theta, alpha, beta, and gamma bands and over the whole frequency range. **g** Frequencies of maximum ERO activity within the delta, theta, alpha, beta, and gamma bands and over the whole frequency range. **h** Grand average resting-state neural activity of alcohol-dependent animals before and ca. one hour after application of psilocybin or LY379268 as means over all channels ± SD. Data is given as power spectral density (PSD) in dB. **i** Resting-state neural activity (as PSD in dB) within the delta, theta, alpha, beta, and gamma bands before and after administration of psilocybin or LY379268. Graphs illustrated in black: alcohol- and drug-naive controls (n = 10), red: alcohol-dependent animals (n = 10), green: alcohol-dependent animals that received a single dose of psilocybin, blue: alcohol-dependent animals that received a single dose of LY379268. Panels b-c, e-g), and i) display data points for all channels of all rats and mean barplots ± 95% confidence interval. Asterisks indicate significant differences between interventions with *p < 0.05, **p < 0.01, ***p < 0.001.

Neurophysiological data revealed a significant effect of long-term alcohol consumption for most neural parameters (Fig. 2) with reduced ERP amplitudes of P1N1 and N1P2 components in alcohol-dependent rats, thus also resulting in earlier peaking of N1 and P2 components compared to naïve controls (Fig. 2a–c, S2, Table S1). In contrast, P2N2 amplitudes were enhanced following long-term alcohol consumption, while N2P3 amplitudes remained similar to controls. Furthermore, alcohol-dependent animals displayed reduced and later peaking ERO activities within delta, theta, alpha and beta bands and over the whole frequency range (Fig. 2d–f, Table S1). Only in the gamma range did long-term alcohol consumption induce an increased oscillatory power. We further observed an elevated activity within higher beta frequency ranges in alcohol-dependent animals while low beta frequencies dominated in naïve controls (Fig. 2g; Table S1). Statistical analyses did not yield an effect of channel location or channel × treatment interaction for any of the parameters.

**Fig. 3:**
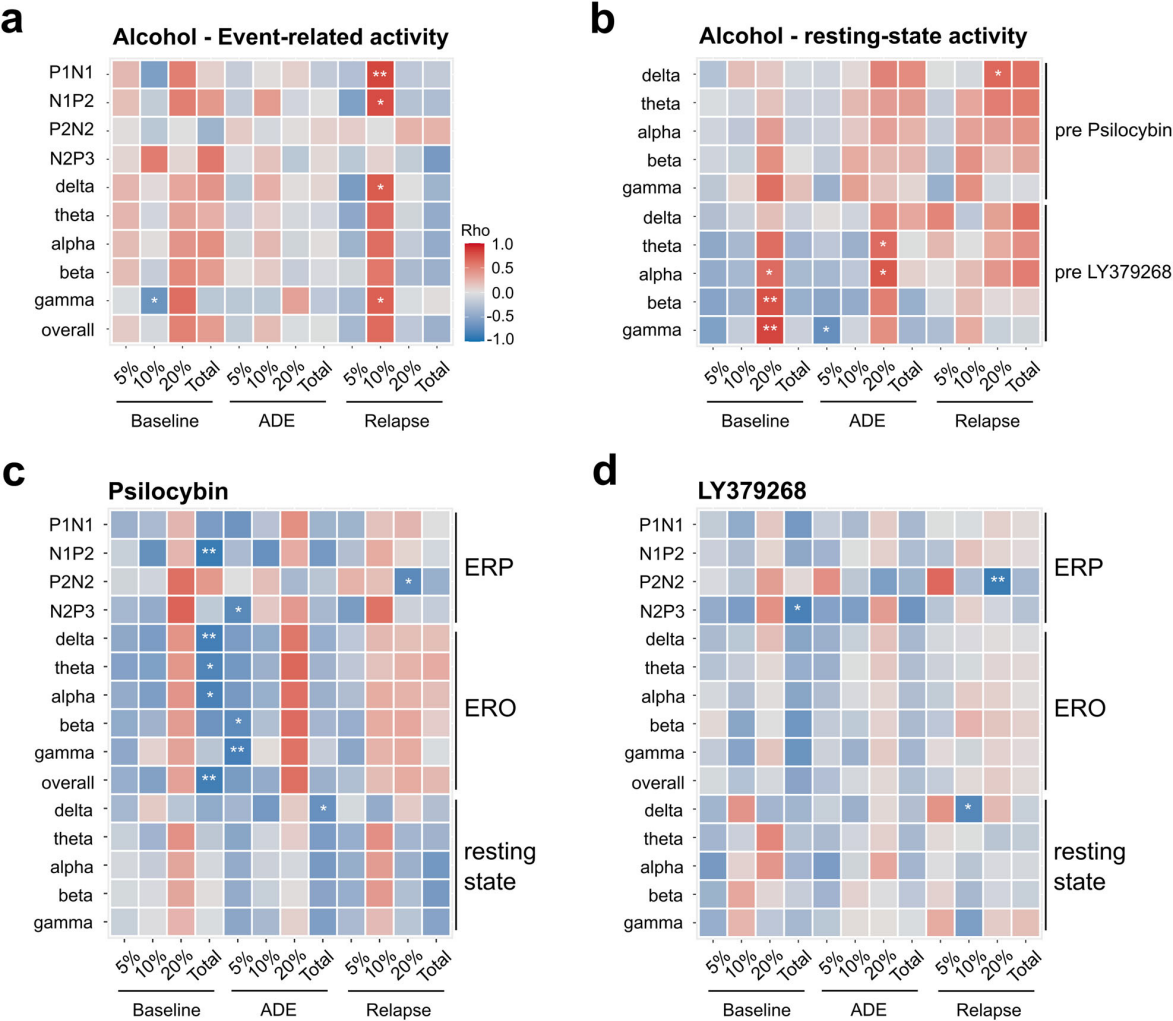
Correlation of electrophysiological activity with alcohol consumption. **a** Spearman correlation of ERP amplitudes and ERO activity (as event-related spectral perturbation, ERSP) and **b** resting-state oscillatory activity (as power spectral density, PSD) with alcohol consumption in alcohol-dependent animals before pharmacological interventions. Partial Spearman correlation of event-related (ERP amplitudes, ERSP) and resting-state activity (PSD) with alcohol consumption following administration of **c** Psilocybin or **d** LY379268, controlled for neural activity acquired under drug-free conditions as given in **a** and **b**. Largely negative (blue colored) correlations (**c**, **d**) illustrate that lower alcohol intake *(*5%, 10% EtOH) at baseline and during the ADE were more responsive to esp. Psilocybin as oppose to largely positive (red colored) correlations following intake of high concentrations (20% EtOH). Electrophysiological activity data used as means over all channels. Alcohol consumption patterns are given as means over the whole experimental period corresponding to baseline (BL) drinking during the last week of a drinking phase, the first day following periods of abstinence (i.e. Alcohol Deprivation Effect (ADE)) and relapse intensities (difference of ADE and previous BL). Rho: Spearman’s correlation coefficient with rho ≥ 0.1 = weak, ≥ 0.4 = moderate, ≥ 0.7 = strong, ≥ 0.9 = very strong correlation. Asterisks indicate significant results with *p < 0.05, **p < 0.01.

Concerning alcohol consumption patterns, P1N1 and N1P2 amplitudes and oscillatory activities of the whole frequency range displayed a strong positive correlation with relapse intensity to 10% alcohol and a moderate positive correlation to baseline consumption of 20% alcohol. In addition, gamma band activity revealed a strong negative correlation with baseline consumption of 10% alcohol (Fig. 3a, Table S2).

Correlation analyses of resting-state oscillatory activities with alcohol consumption before substance application most consistently revealed moderate to strong positive correlations with consumption of 20% alcohol at baseline and after abstinence. This relation was also observed for delta, theta, and alpha waves, with total alcohol relapse intensity primarily attributed again to 20% alcohol. In addition, gamma activity displayed a moderate negative correlation with post-deprivation consumption of 5% alcohol (Fig. 3b, Table S3).

### Pharmacological modulation of neural activity in alcohol-dependent rats

After successfully investigating the electrophysiological changes in alcohol-dependent rats, we wanted to examine whether pharmacological interventions counteract these neuropathological changes. We previously demonstrated that pharmacotreatment with psilocybin and LY379268 reduces alcohol intake following abstinence, thereby significantly counteracting the ADE (Fig. 1c) [44, 58]. We build upon this work and investigated the neurophysiological effects induced by acute psilocybin and LY379268.

#### Psilocybin

Following an acute application of 2.5 mg/kg psilocybin in alcohol-detoxified rats, we observed a partial decrease in resting-state oscillatory activities, predominantly in delta and theta bands, with no effect of channel location or channel × treatment interaction (Fig. 2h, i, upper panels, Table S4). Bandpowers after vs. before drug administration were positively correlated with moderate to strong effect sizes (Table S5). Correlation with alcohol consumption revealed most consistently a moderate, negative correlation of post-administration resting-state bandpowers with total alcohol consumption after abstinence and corresponding relapse intensity and a moderate, positive correlation of theta, alpha, beta and gamma band activities with baseline consumption of 20% alcohol (Fig. 3b, Table S6).

After administration of psilocybin, ERPs of alcohol-dependent rats revealed increased amplitudes of P1N1, N1P2 and N2P3 and reduced peak latencies of N2 and P3 components. (Fig. 2a, b, Table S7). The manifestation of these ERPs was similar to those observed in naïve rats and even showed slightly higher and earlier peaking amplitudes (Fig. 2a, b, Table S8). In contrast to resting-state oscillations, EROs displayed elevated bandpowers over the whole frequency range (Fig. 2d, e, Table S7). We additionally observed a shift from maximum beta powers to lower frequency ranges after administering psilocybin (Fig. 2d, g, Table S7). Compared to naïve controls, ERO activity differed predominantly in the gamma frequency range, revealing increased and earlier peaking bandpowers (Fig. 2e, f, Table S8). Correlation analyses of event-related activity following psilocybin administration vs. initial recordings without drug application revealed a moderate negative correlation for P2N2 amplitudes, while all other ERPs and EROs were positively correlated with varying effect sizes (Table S9). Regarding alcohol consumption patterns, we observed a positive correlation between post-administration ERPs and EROs and drinking amounts of 20% alcohol at baseline and following deprivation and a negative correlation with total baseline alcohol intake and consumption of 5% alcohol following abstinence (Fig. 3c, Table S10).

#### LY379268

The administration of 1 mg/kg LY379268 decreased resting-state oscillatory activity within all frequency bands and at all electrode sites with medium to large effect sizes (Fig. 2h, i, bottom panels, Table S11). Bandpowers after vs. before drug application were positively correlated (Table S12), whereas the correlation of post-administration bandpowers with alcohol consumption patterns displayed only a weak connection overall (Fig. 3d, Table S13).

Following administration of LY379268, ERPs revealed increased P1N1 and N1P2 and decreased P2N2 amplitudes (Fig. 2a, b; Table S14) and an elevated ERO activity (Fig. 2d, e; Table S14). As with psilocybin, beta frequencies dominated at lower frequency ranges compared to the non-treated condition (Fig. 2g, Table S14). Compared to naïve controls, ERP amplitudes were in the same range (Fig. 2a, b, Table S15), while EROs displayed an increased and earlier peaking gamma activity under the influence of LY379268 (Fig. 2g, Table S15). Event-related activity following LY379268 administration displayed weak positive correlations with initial recordings without drug application (Table S16) and weaker connections to alcohol consumption patterns than those recorded under the influence of psilocybin, but indicates a similar negative relation to total alcohol intake at baseline and specifically with the relapse intensity to 20% alcohol in case of the P2N2 component (Fig. 2d, Table S17).

## Discussion

In the present study, we used an established animal model of compulsive alcohol consumption and relapse behavior and a new neuroprosthetic interface to record, modulate and classify potential electrophysiological biomarkers to reveal i) neuroelectric changes in the prefrontal cortex in alcohol-dependent rats and ii) their reversal via application of the psychedelic psilocybin and the mGluR2 agonist LY379268.

Alcohol-dependent rats displayed reduced ERP amplitudes of P1N1 and N1P2 components and decreased and later peaking ERO activity within the delta and theta frequency bands, indicating deficiencies in sensory gating and early attentive filtering [17, 19, 59]. However, in the gamma range, chronic alcohol intake induced an increased oscillatory power, which has been related to enhanced extracellular glutamate concentrations [60, 61]. We further observed a dominance within higher beta frequency ranges in alcohol-dependent rats compared to naïve controls with maximum band powers in low beta frequencies. In patients with AUD, abnormal frontal high beta and gamma activities have been associated with an impaired working memory system and deficient top-down processing known to provoke reduced executive control and inhibition [62, 63] underlying relapse behavior. Manifestation of ERPs and EROs showed a diametrically opposite correlation with baseline drinking compared to the relapse-like increase in alcohol consumption following abstinence, suggesting that relapse-like drinking and baseline alcohol consumption are controlled through different neural mechanisms.

Disturbed sensory gating, as reflected by P1N1 and N1P2 deficiencies, has been correlated with perceptual aberrations and the proneness to develop psychotic symptoms [64]. Acute application of psilocybin and LY379268 increased amplitudes of P1N1 and N1P2 components in our alcohol-dependent rats and, in the same time range, elevated bandpowers over the entire frequency range. In addition, we show that both pharmaco-treatments shifted maximum beta powers from mid/high beta ranges to lower frequencies. Beta oscillations have so far been examined predominantly with respect to sensorimotor function [65] rarely distinguishing between low (12 - 15 Hz), mid (15 - 18 Hz) and high beta frequencies ( > 18 Hz). Our findings thus provide novel insights into the involvement of beta subunits in cognition, supporting current associations of mid/high beta ranges with stress, anxiety and paranoia, and lower frequencies reflecting increased attention and self-regulation [66–68].

Enhanced ERP amplitudes with additionally shorter latencies following psilocybin administration, exceeding those observed in controls, suggest a neuroenhancing effect, by which psilocybin does not only re-instate ERP amplitudes but promotes faster signal processing. In contrast, the reduced peak latencies observed prior to pharmaco-treatment are a direct consequence of smaller ERP amplitudes following prolonged alcohol consumption and thus indicate disturbed sensory processing.

Both pharmacological treatments further restored a pronounced P1-N1-P2 complex comparable to naïve controls that directly disembogued into the P3 component rather than forming a marked N2. A marked N2P2 component was a distinct feature of untreated alcohol-dependent rats. Higher N2 amplitudes have been related to increased task difficulty and effort necessary to inhibit behavior, as in NoGo responses [69]. An elevated N2 component in alcohol-dependent rats might, therefore, indicate an inappropriate activation of mental resources not necessary for the simple passive listening task applied here. In contrast, a small N2 might further emphasize the facilitation of sensory information processing in response to both applied pharmacological interventions.

We noted that enhanced and early peaking event-related gamma activity in alcohol-dependent rats was not antagonist by pharmaco-treatment. Event-related gamma oscillations have been distinguished into sensory and cognitive gamma responses with the former occurring about 100 ms post-stimulus while the latter peak at about 300 ms [70]. Later peaking cognitive gamma responses dominated in the control individuals. Cognitive gamma oscillations have been linked to mechanisms of synaptic plasticity and supposedly reflect the coordinated activity of neuronal assemblies supporting memory formation, effective learning, and focused attention [71]. In contrast, in alcohol-dependent animals, gamma activity is more restricted to early-peaking sensory responses underlining once more the chronic alcohol-induced neural deficits in higher-order cognitive function. Although psilocybin was able to significantly increase the N2P3 amplitude, cognitive gamma responses were not influenced by either treatment as seen by unaffected gamma latencies. In line with our findings of the most pronounced increase in amplitudes of early ERPs the applied pharmaco-treatments thus targeted predominantly sensory processing.

However, cognitive control might be supported by the increase in event-related oscillatory activity within lower frequency ranges of the delta, theta, and alpha bands. Their synchronization predicts P1N1 latency and amplitude and contributes to forming the P3 component [17, 21]. Of note, in humans, these frequency bands are related to relaxation, mindfulness and creativity [66]. Finally, and in line with previous preclinical studies, psilocybin and LY379268 decreased resting-state activity over the entire frequency range, mitigating neural hyperarousal in our alcohol-dependent rats, though this effect was not as pronounced as previously seen in healthy rats [72, 73]. Since disturbances in both, resting state and event-related neural activity, have been related to increased relapse probability, a state change through pharmaco-treatment such as psilocybin (independently if it is through a reduction of global resting state activity or strengthening of low-frequency event-related activity or both) may assist mindfulness-based interventions for addictive behaviors shown to be beneficial in reducing substance misuse and craving [74, 75].

5-HT_2A_R and mGluR2 have been shown to physiologically interact antagonistically to ensure correct glutamate exocytosis [76]. Therefore, it seems surprising that in this study, we found similar effects of the administration of psilocybin and LY379268 on electrophysiological brain activity. One possible explanation might be the aforementioned dysfunction of mGluR2 often observed in patients with AUD. In addition, a recent study in mice displaying pharmacologically induced deficiencies in auditory ERO activity confirmed the beneficial effect of LY379268 [77]. Further experiments should consider the co-administration of these two compounds as well as the administration of mGluR2 and 5-HT_2A_R antagonists in combination with psilocybin and LY379268, respectively. These results will help to deepen our understanding of the receptor-receptor crosstalk and its effects on brain activity.

Correlation analysis further revealed that the changes induced by pharmacological treatment in resting-state and event-related activity depend on both individual neural activity states without drug application and alcohol consumption patterns. The ameliorative effects of pharmacological treatments on ERPs and EROs were more pronounced the lower the alcohol intake had been, as indicated by largely negative correlations. Interestingly, these findings were restricted to low and moderately concentrated alcohol solutions, while specifically psilocybin-induced enhancements of neural activity were positively correlated with the consumption of 20% alcohol. Differential neuronal activity as measured here through our neuroprosthetic interface, therefore, reflects both individual alcohol consumption habits and may predict responsiveness to pharmacotreatment.

To summarize, we have employed a custom-made ECoG interface to acquire neurophysiological impairments induced by chronic alcohol use and to monitor their restoration to mechanism-based pharmacological interventions. Prospectively, neural implants would also offer the possibility for local drug application, enabling a more precise and controllable drug delivery with reduced side effects compared to systemic application [78]. The evaluated electrophysiological parameters have been related to the onset and persistence of clinical symptoms and may predict the clinical trajectory in individuals [29, 30]. In conjunction with the ADE rat model, which mimics key characteristics of AUD-related pathophysiology and behavioral deficits, this approach provides a powerful translational toolbox showcased by investigating the effects of administering psilocybin and LY379268 which ultimately support recent suggestions of a 5-HT_2A_R-mGluR2-based approach to therapeutically target AUD.

## Supplementary information

Supplemental Figures

Supplemental Tables

## Data Availability

All data needed to evaluate the conclusions in the paper are present in the paper and/or the Supplementary Materials. Employed code can be found on *GitHub* (https://github.com/habeltb/Multimodal_ECoG_ratPFC_alcoholism).
