# Supplementary material for: Prefrontal electrophysiological biomarkers and mechanism-based drug effects in a rat model of alcohol addiction
Source: Transl Psychiatry. 2024 Dec 5;14:486. doi: 10.1038/s41398-024-03189-z (PMC11621398; doi:10.1038/s41398-024-03189-z)
Supplement: Supplementary file 1 — Supplemental Figures [file 41398_2024_3189_MOESM1_ESM.pdf]

Supplementary information

Prefrontal Electrophysiological Biomarkers and Mechanism-Based Drug Effects in a Rat Model of Alcohol Addiction

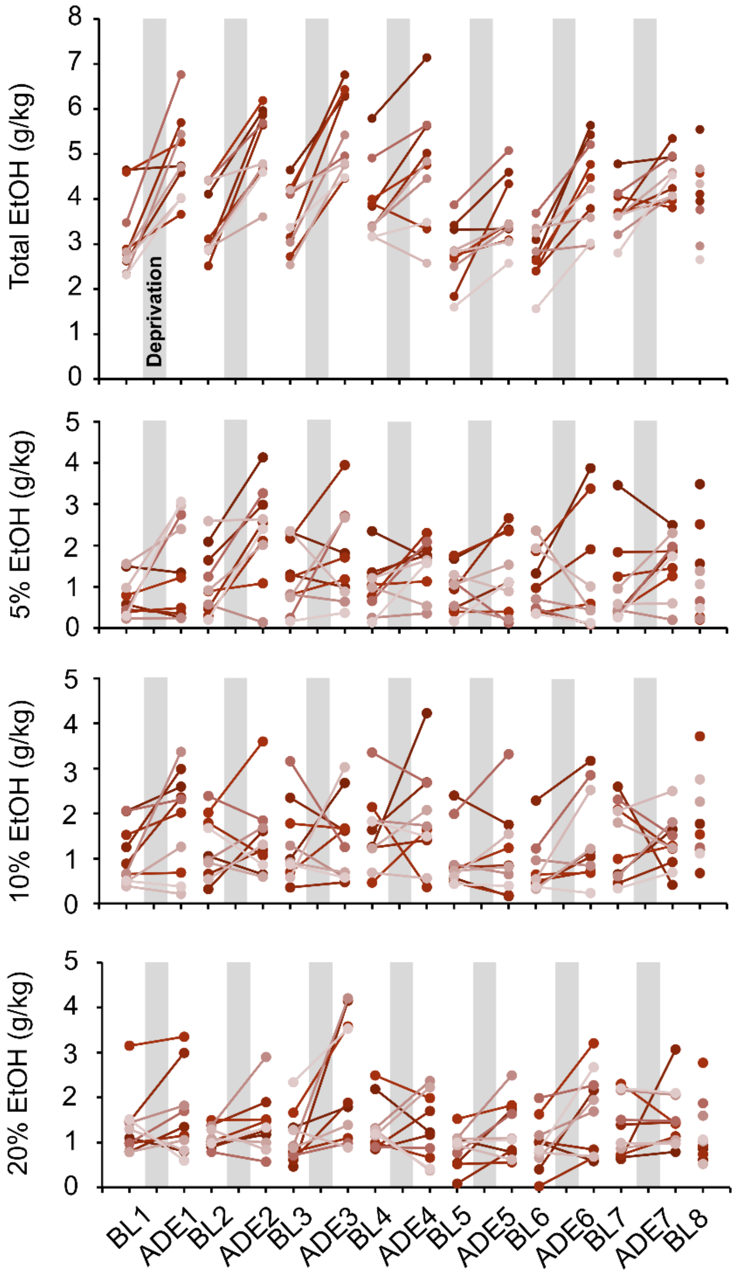

Fig. S1 Individual long-term alcohol consumption throughout the experimental timeline. In alternating cycles, animals had access to either alcohol solutions of 5 %, 10 % and 20 % ethanol besides water or to water alone (deprivation, grey). Data are presented as individual data points of ten animals of pure EtOH in g per kg during the last week of a drinking phase (baselines, BL) and on the first day following periods of abstinence (i.e. Alcohol Deprivation Effect (ADE)). Solid lines between ADEs and previous BLs indicate relapse intensities, i.e. changes in consumption behaviour following alcohol deprivation.

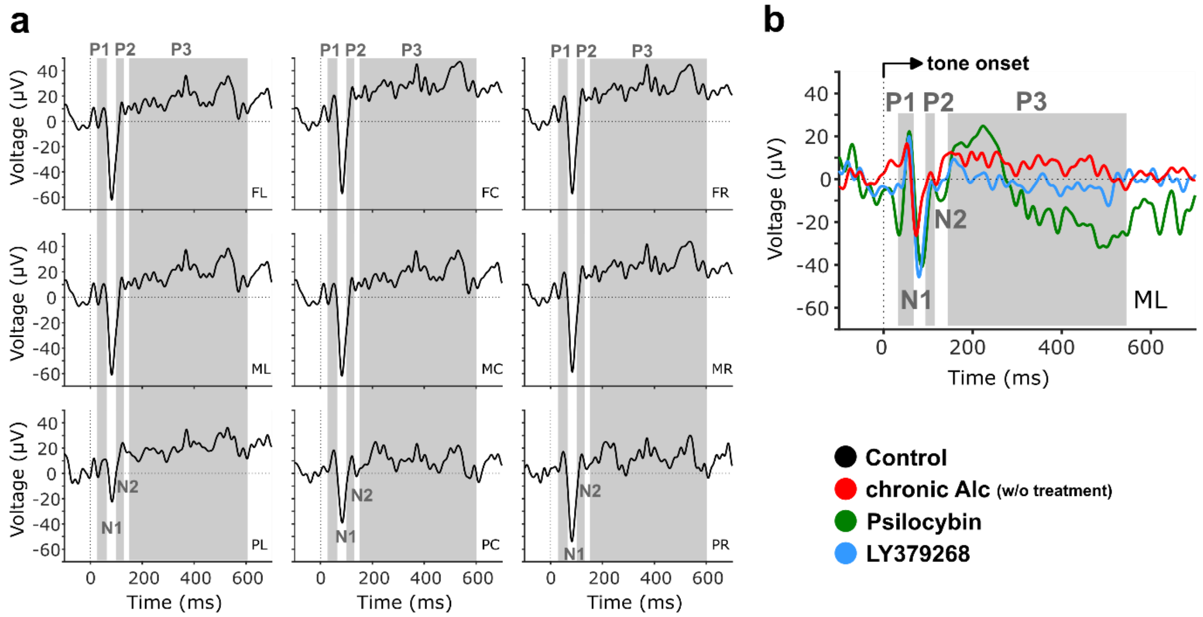

Fig. S2 Representative single-subject average deviant-minus-standard auditory event-related potential curves. a) Data of one alcohol- and drug-naïve animal of all channels indicating similar recordings at all electrode sites. **b)** Data of one channel of one alcohol-dependent animal before and following treatment with Psilocybin or LY379268. Time intervals for detection of P1, P2, and P3 peak amplitudes in these animals are shaded in grey with white gaps in between indicating time intervals of N1 and N2 components. Channels are named according to their location on the neuroprosthetic device as frontal-left (FL), frontocentral (FC), frontal-right (FR), medial-left (ML), mediocentral (MC), medial-right (MR), parietal-left (PL), parietocentral (PC), parietal-right (PR).
