# Supplementary material for: Prefrontal electrophysiological biomarkers and mechanism-based drug effects in a rat model of alcohol addiction
Source: Transl Psychiatry. 2024 Dec 5;14:486. doi: 10.1038/s41398-024-03189-z (PMC11621398; doi:10.1038/s41398-024-03189-z)
Supplement: Supplementary file 2 — Supplemental Tables [file 41398_2024_3189_MOESM2_ESM.pdf]

## *Supplementary information*

# Prefrontal Electrophysiological Biomarkers and Mechanism-Based Drug Effects in a Rat Model of Alcohol Addiction

**Table S1** Impact of chronic alcohol intake in the ADE rat model on event-related neural activity compared to alcohol-naïve controls.

| Parameter | df | F | p | $\eta_p^2$ |
|---|---|---|---|---|
| LatP1 | 1, 152 | 10.719 | ***0.001*** | 0.066 |
| LatN1 | 1, 152 | 65.965 | ***0.000*** | 0.303 |
| LatP2 | 1, 152 | 19.335 | ***0.000*** | 0.113 |
| LatN2 | 1, 152 | 0.011 | 0.916 | 0.000 |
| LatP3 | 1, 152 | 0.084 | 0.773 | 0.001 |
| AmplP1N1 | 1, 143 | 13.351 | ***0.000*** | 0.085 |
| AmplN1P2 | 1, 143 | 3.946 | ***0.049*** | 0.027 |
| AmplP2N2 | 1, 143 | 33.688 | ***0.000*** | 0.191 |
| AmplN2P3 | 1, 143 | 3.175 | 0.077 | 0.022 |
| deltaPower_dB | 1, 143 | 13.232 | ***0.000*** | 0.085 |
| thetaPower_dB | 1, 143 | 16.530 | ***0.000*** | 0.104 |
| alphaPower_dB | 1, 143 | 17.290 | ***0.000*** | 0.108 |
| betaPower_dB | 1, 143 | 10.947 | ***0.001*** | 0.071 |
| gammaPower_dB | 1, 143 | 20.644 | ***0.000*** | 0.126 |
| TotalPower_dB | 1, 143 | 16.027 | ***0.000*** | 0.101 |
| Time_deltaPower_max | 1, 143 | 4.853 | ***0.029*** | 0.033 |
| Freq_deltaPower_max | 1, 143 | 5.647 | ***0.019*** | 0.038 |
| Time_thetaPower_max | 1, 143 | 3.704 | 0.056 | 0.025 |
| Freq_thetaPower_max | 1, 143 | 3.353 | 0.069 | 0.023 |
| Time_alphaPower_max | 1, 143 | 1.169 | 0.281 | 0.008 |
| Freq_alphaPower_max | 1, 143 | 0.252 | 0.617 | 0.002 |
| Time_betaPower_max | 1, 143 | 5.284 | ***0.023*** | 0.036 |
| Freq_betaPower_max | 1, 143 | 50.909 | ***0.000*** | 0.263 |
| Time_gammaPower_max | 1, 143 | 28.216 | ***0.000*** | 0.165 |
| Freq_gammaPower_max | 1, 143 | 19.872 | ***0.000*** | 0.122 |
| Time_TotalPower_max | 1, 143 | 0.148 | 0.701 | 0.001 |
| Freq_TotalPower_max | 1, 143 | 13.526 | ***0.000*** | 0.086 |

ANOVA – main effect of treatment (no effect of channel location or treatment*channel interaction), df: degrees of freedom, $\eta_p^2$: effect size partial eta squared with $\eta_p^2 \geq 0.01$ = small, $\eta_p^2 \geq 0.06$ = medium and $\eta_p^2 > 0.14$ = large effects. Significant *p*-values given in bold, italic.

**Table S2** Correlation of event-related activity with alcohol consumption in the ADE rat model.

| Parameter | Alcohol consumption | *ρ* | p |
|---|---|---|---|
| AmplP1N1 | BL_Alc5 | 0.217 | 0.576 |
| | BL_Alc10 | -0.567 | 0.112 |
| | BL_Alc20 | 0.533 | 0.139 |
| | BL_TotalAlc | 0.083 | 0.831 |
| | ADE_Alc5 | -0.150 | 0.700 |
| | ADE_Alc10 | 0.017 | 0.966 |
| | ADE_Alc20 | 0.083 | 0.831 |
| | ADE_TotalAlc | -0.183 | 0.637 |
| | Relapse_Alc5 | -0.267 | 0.488 |
| | Relapse_Alc10 | 0.812 | ***0.008*** |
| | Relapse_Alc20 | -0.200 | 0.606 |
| | Relapse_TotalAlc | -0.183 | 0.637 |
| AmplN1P2 | BL_Alc5 | 0.183 | 0.637 |
| | BL_Alc10 | -0.167 | 0.668 |
| | BL_Alc20 | 0.533 | 0.139 |
| | BL_TotalAlc | 0.400 | 0.286 |
| | ADE_Alc5 | -0.183 | 0.637 |
| | ADE_Alc10 | 0.383 | 0.308 |
| | ADE_Alc20 | -0.083 | 0.831 |
| | ADE_TotalAlc | 0.000 | 1.000 |
| | Relapse_Alc5 | -0.550 | 0.125 |
| | Relapse_Alc10 | 0.787 | ***0.012*** |
| | Relapse_Alc20 | -0.250 | 0.516 |
| | Relapse_TotalAlc | -0.300 | 0.433 |
| AmplP2N2 | BL_Alc5 | 0.017 | 0.966 |
| | BL_Alc10 | -0.217 | 0.576 |
| | BL_Alc20 | 0.000 | 1.000 |
| | BL_TotalAlc | -0.383 | 0.308 |
| | ADE_Alc5 | 0.117 | 0.765 |
| | ADE_Alc10 | -0.067 | 0.865 |
| | ADE_Alc20 | 0.017 | 0.966 |
| | ADE_TotalAlc | 0.083 | 0.831 |
| | Relapse_Alc5 | 0.100 | 0.798 |
| | Relapse_Alc10 | 0.000 | 1.000 |
| | Relapse_Alc20 | 0.250 | 0.516 |
| | Relapse_TotalAlc | 0.250 | 0.516 |
| AmplN2P3 | BL_Alc5 | 0.067 | 0.865 |
| | BL_Alc10 | 0.550 | 0.125 |
| | BL_Alc20 | 0.067 | 0.865 |
| | BL_TotalAlc | 0.567 | 0.112 |
| | ADE_Alc5 | -0.050 | 0.898 |
| | ADE_Alc10 | 0.167 | 0.668 |
| | ADE_Alc20 | -0.217 | 0.576 |
| | ADE_TotalAlc | 0.050 | 0.898 |
| | Relapse_Alc5 | -0.217 | 0.576 |
| | Relapse_Alc10 | 0.075 | 0.847 |
| | Relapse_Alc20 | -0.167 | 0.668 |
| | Relapse_TotalAlc | -0.600 | 0.088 |
| deltaPower_dB | BL_Alc5 | 0.233 | 0.546 |
| | BL_Alc10 | 0.033 | 0.932 |
| | BL_Alc20 | 0.317 | 0.406 |
| | BL_TotalAlc | 0.417 | 0.265 |
| | ADE_Alc5 | -0.217 | 0.576 |
| | ADE_Alc10 | 0.267 | 0.488 |
| | ADE_Alc20 | 0.017 | 0.966 |
| | ADE_TotalAlc | 0.067 | 0.865 |
| | Relapse_Alc5 | -0.583 | 0.099 |
| | Relapse_Alc10 | 0.720 | ***0.029*** |
| | Relapse_Alc20 | 0.000 | 1.000 |
| | Relapse_TotalAlc | -0.383 | 0.308 |

| Parameter | Alcohol consumption | *ρ* | p |
|---|---|---|---|
| thetaPower_dB | BL_Alc5 | 0.233 | 0.546 |
| | BL_Alc10 | -0.083 | 0.831 |
| | BL_Alc20 | 0.333 | 0.381 |
| | BL_TotalAlc | 0.267 | 0.488 |
| | ADE_Alc5 | -0.133 | 0.732 |
| | ADE_Alc10 | 0.100 | 0.798 |
| | ADE_Alc20 | -0.067 | 0.865 |
| | ADE_TotalAlc | -0.083 | 0.831 |
| | Relapse_Alc5 | -0.467 | 0.205 |
| | Relapse_Alc10 | 0.636 | 0.066 |
| | Relapse_Alc20 | -0.100 | 0.798 |
| | Relapse_TotalAlc | -0.450 | 0.224 |
| alphaPower_dB | BL_Alc5 | 0.200 | 0.606 |
| | BL_Alc10 | 0.017 | 0.966 |
| | BL_Alc20 | 0.417 | 0.265 |
| | BL_TotalAlc | 0.450 | 0.224 |
| | ADE_Alc5 | -0.100 | 0.798 |
| | ADE_Alc10 | 0.217 | 0.576 |
| | ADE_Alc20 | -0.100 | 0.798 |
| | ADE_TotalAlc | 0.017 | 0.966 |
| | Relapse_Alc5 | -0.383 | 0.308 |
| | Relapse_Alc10 | 0.628 | 0.070 |
| | Relapse_Alc20 | -0.183 | 0.637 |
| | Relapse_TotalAlc | -0.450 | 0.224 |
| betaPower_dB | BL_Alc5 | 0.200 | 0.606 |
| | BL_Alc10 | -0.150 | 0.700 |
| | BL_Alc20 | 0.483 | 0.187 |
| | BL_TotalAlc | 0.383 | 0.308 |
| | ADE_Alc5 | 0.017 | 0.966 |
| | ADE_Alc10 | 0.117 | 0.765 |
| | ADE_Alc20 | -0.150 | 0.700 |
| | ADE_TotalAlc | -0.050 | 0.898 |
| | Relapse_Alc5 | -0.100 | 0.798 |
| | Relapse_Alc10 | 0.569 | 0.110 |
| | Relapse_Alc20 | -0.317 | 0.406 |
| | Relapse_TotalAlc | -0.400 | 0.286 |
| gammaPower_dB | BL_Alc5 | -0.033 | 0.932 |
| | BL_Alc10 | -0.667 | ***0.050*** |
| | BL_Alc20 | 0.633 | 0.067 |
| | BL_TotalAlc | -0.217 | 0.576 |
| | ADE_Alc5 | -0.217 | 0.576 |
| | ADE_Alc10 | -0.217 | 0.576 |
| | ADE_Alc20 | 0.350 | 0.356 |
| | ADE_TotalAlc | -0.200 | 0.606 |
| | Relapse_Alc5 | -0.333 | 0.381 |
| | Relapse_Alc10 | 0.695 | ***0.038*** |
| | Relapse_Alc20 | -0.033 | 0.932 |
| | Relapse_TotalAlc | 0.017 | 0.966 |
| TotalPower_dB | BL_Alc5 | 0.117 | 0.765 |
| | BL_Alc10 | -0.067 | 0.865 |
| | BL_Alc20 | 0.517 | 0.154 |
| | BL_TotalAlc | 0.383 | 0.308 |
| | ADE_Alc5 | -0.167 | 0.668 |
| | ADE_Alc10 | 0.200 | 0.606 |
| | ADE_Alc20 | -0.033 | 0.932 |
| | ADE_TotalAlc | -0.017 | 0.966 |
| | Relapse_Alc5 | -0.333 | 0.381 |
| | Relapse_Alc10 | 0.644 | 0.061 |
| | Relapse_Alc20 | -0.200 | 0.606 |
| | Relapse_TotalAlc | -0.400 | 0.286 |

*ρ*: Spearman´s correlation coefficient *rho* with $|\rho| \geq 0.1$ = small, $|\rho| \geq 0.3$ = medium and $|\rho| > 0.5$ = large effects. Significant *p*-values given in bold, italic.

**Table S3** Correlation of resting state oscillatory activity (prior to application of Psilocybin or LY379268) with alcohol consumption in the ADE rat model.

| Parameter | Alcohol consumption | *ρ* | p |
|---|---|---|---|
| deltaPower_dB_prePsi | BL_Alc5 | -0.261 | 0.467 |
| | BL_Alc10 | 0.188 | 0.602 |
| | BL_Alc20 | 0.127 | 0.726 |
| | BL_TotalAlc | -0.079 | 0.829 |
| | ADE_Alc5 | -0.091 | 0.803 |
| | ADE_Alc10 | 0.042 | 0.907 |
| | ADE_Alc20 | 0.515 | 0.128 |
| | ADE_TotalAlc | 0.467 | 0.174 |
| | Relapse_Alc5 | -0.018 | 0.960 |
| | Relapse_Alc10 | -0.067 | 0.854 |
| | Relapse_Alc20 | 0.648 | ***0.043*** |
| | Relapse_TotalAlc | 0.608 | 0.062 |
| thetaPower__dB_prePsi | BL_Alc5 | -0.030 | 0.934 |
| | BL_Alc10 | -0.091 | 0.802 |
| | BL_Alc20 | 0.164 | 0.651 |
| | BL_TotalAlc | -0.127 | 0.726 |
| | ADE_Alc5 | -0.127 | 0.726 |
| | ADE_Alc10 | 0.224 | 0.533 |
| | ADE_Alc20 | 0.370 | 0.293 |
| | ADE_TotalAlc | 0.382 | 0.276 |
| | Relapse_Alc5 | -0.164 | 0.651 |
| | Relapse_Alc10 | 0.316 | 0.374 |
| | Relapse_Alc20 | 0.539 | 0.108 |
| | Relapse_TotalAlc | 0.553 | 0.097 |
| alphaPower_dB_prePsi | BL_Alc5 | -0.091 | 0.803 |
| | BL_Alc10 | -0.195 | 0.590 |
| | BL_Alc20 | 0.370 | 0.293 |
| | BL_TotalAlc | -0.152 | 0.676 |
| | ADE_Alc5 | -0.127 | 0.726 |
| | ADE_Alc10 | 0.127 | 0.726 |
| | ADE_Alc20 | 0.345 | 0.328 |
| | ADE_TotalAlc | 0.248 | 0.489 |
| | Relapse_Alc5 | -0.164 | 0.651 |
| | Relapse_Alc10 | 0.353 | 0.318 |
| | Relapse_Alc20 | 0.370 | 0.293 |
| | Relapse_TotalAlc | 0.426 | 0.220 |
| betaPower_dB_prePsi | BL_Alc5 | -0.091 | 0.803 |
| | BL_Alc10 | -0.146 | 0.688 |
| | BL_Alc20 | 0.455 | 0.187 |
| | BL_TotalAlc | -0.006 | 0.987 |
| | ADE_Alc5 | -0.176 | 0.627 |
| | ADE_Alc10 | 0.297 | 0.405 |
| | ADE_Alc20 | 0.224 | 0.533 |
| | ADE_TotalAlc | 0.236 | 0.511 |
| | Relapse_Alc5 | -0.248 | 0.489 |
| | Relapse_Alc10 | 0.432 | 0.213 |
| | Relapse_Alc20 | 0.188 | 0.603 |
| | Relapse_TotalAlc | 0.328 | 0.354 |
| gammaPower_dB_prePsi | BL_Alc5 | -0.212 | 0.556 |
| | BL_Alc10 | 0.061 | 0.868 |
| | BL_Alc20 | 0.612 | 0.060 |
| | BL_TotalAlc | 0.176 | 0.627 |
| | ADE_Alc5 | -0.394 | 0.260 |
| | ADE_Alc10 | 0.358 | 0.310 |
| | ADE_Alc20 | 0.164 | 0.651 |
| | ADE_TotalAlc | 0.067 | 0.855 |
| | Relapse_Alc5 | -0.418 | 0.229 |
| | Relapse_Alc10 | 0.450 | 0.192 |
| | Relapse_Alc20 | -0.067 | 0.855 |
| | Relapse_TotalAlc | -0.049 | 0.894 |

| Parameter | Alcohol consumption | *ρ* | p |
|---|---|---|---|
| deltaPower_dB_preLY | BL_Alc5 | -0.273 | 0.446 |
| | BL_Alc10 | -0.109 | 0.763 |
| | BL_Alc20 | 0.176 | 0.627 |
| | BL_TotalAlc | -0.127 | 0.726 |
| | ADE_Alc5 | 0.018 | 0.960 |
| | ADE_Alc10 | -0.091 | 0.803 |
| | ADE_Alc20 | 0.467 | 0.174 |
| | ADE_TotalAlc | 0.309 | 0.385 |
| | Relapse_Alc5 | 0.503 | 0.138 |
| | Relapse_Alc10 | -0.201 | 0.578 |
| | Relapse_Alc20 | 0.358 | 0.310 |
| | Relapse_TotalAlc | 0.608 | 0.062 |
| thetaPower_dB_preLY | BL_Alc5 | -0.479 | 0.162 |
| | BL_Alc10 | -0.328 | 0.354 |
| | BL_Alc20 | 0.624 | 0.054 |
| | BL_TotalAlc | -0.370 | 0.293 |
| | ADE_Alc5 | -0.200 | 0.580 |
| | ADE_Alc10 | -0.394 | 0.260 |
| | ADE_Alc20 | 0.636 | ***0.048*** |
| | ADE_TotalAlc | -0.018 | 0.960 |
| | Relapse_Alc5 | 0.224 | 0.533 |
| | Relapse_Alc10 | -0.006 | 0.987 |
| | Relapse_Alc20 | 0.297 | 0.405 |
| | Relapse_TotalAlc | 0.444 | 0.199 |
| alphaPower_dB_preLY | BL_Alc5 | -0.442 | 0.200 |
| | BL_Alc10 | -0.334 | 0.345 |
| | BL_Alc20 | 0.636 | ***0.048*** |
| | BL_TotalAlc | -0.321 | 0.365 |
| | ADE_Alc5 | -0.333 | 0.347 |
| | ADE_Alc10 | -0.200 | 0.580 |
| | ADE_Alc20 | 0.733 | ***0.016*** |
| | ADE_TotalAlc | 0.091 | 0.803 |
| | Relapse_Alc5 | 0.018 | 0.960 |
| | Relapse_Alc10 | 0.195 | 0.590 |
| | Relapse_Alc20 | 0.406 | 0.244 |
| | Relapse_TotalAlc | 0.541 | 0.106 |
| betaPower_dB_preLY | BL_Alc5 | -0.515 | 0.128 |
| | BL_Alc10 | -0.407 | 0.243 |
| | BL_Alc20 | 0.770 | ***0.009*** |
| | BL_TotalAlc | -0.418 | 0.229 |
| | ADE_Alc5 | -0.491 | 0.150 |
| | ADE_Alc10 | -0.442 | 0.200 |
| | ADE_Alc20 | 0.527 | 0.117 |
| | ADE_TotalAlc | -0.418 | 0.229 |
| | Relapse_Alc5 | -0.067 | 0.855 |
| | Relapse_Alc10 | 0.201 | 0.578 |
| | Relapse_Alc20 | 0.030 | 0.934 |
| | Relapse_TotalAlc | 0.079 | 0.828 |
| gammaPower_dB_preLY | BL_Alc5 | -0.552 | 0.098 |
| | BL_Alc10 | -0.158 | 0.663 |
| | BL_Alc20 | 0.818 | ***0.004*** |
| | BL_TotalAlc | -0.127 | 0.726 |
| | ADE_Alc5 | -0.697 | ***0.025*** |
| | ADE_Alc10 | -0.091 | 0.803 |
| | ADE_Alc20 | 0.455 | 0.187 |
| | ADE_TotalAlc | -0.358 | 0.310 |
| | Relapse_Alc5 | -0.297 | 0.405 |
| | Relapse_Alc10 | 0.298 | 0.403 |
| | Relapse_Alc20 | -0.139 | 0.701 |
| | Relapse_TotalAlc | -0.103 | 0.776 |

*ρ*: Spearman´s correlation coefficient *rho* with $|\rho| \geq 0.1$ = small, $|\rho| \geq 0.3$ = medium and $|\rho| > 0.5$ = large effects. Significant *p*-values given in bold, italic.

**Table S4** Impact of psilocybin on resting state oscillator activity in the ADE rat model.

| **Parameter** | **df** | **F** | **p** | $\eta_p^2$ |
|---|---|---|---|---|
| deltaPower_dB | 1, 75 | 5.452 | ***0.022*** | 0.068 |
| thetaPower_dB | 1, 75 | 5.327 | ***0.024*** | 0.066 |
| alphaPower_dB | 1, 75 | 3.594 | 0.062 | 0.046 |
| betaPower_dB | 1, 75 | 1.382 | 0.243 | 0.018 |
| gammaPower_dB | 1, 75 | 2.983 | 0.088 | 0.038 |

ANOVA – main effect of treatment (no effect of channel location or treatment*channel interaction), df: degrees of freedom, $\eta_p^2$: effect size partial eta squared with $\eta_p^2 \geq 0.01$ = small, $\eta_p^2 \geq 0.06$ = medium and $\eta_p^2 > 0.14$ = large effects. Significant *p*-values given in bold, italic.

**Table S5** Correlation of resting state oscillatory activity after vs. before psilocybin application in the ADE rat model.

| **Parameter** | ***ρ*** | **p** |
|---|---|---|
| deltaPower_dB | -0.539 | 0.108 |
| thetaPower_dB | -0.164 | 0.651 |
| alphaPower_dB | 0.200 | 0.580 |
| betaPower_dB | 0.224 | 0.533 |
| gammaPower_dB | 0.697 | ***0.025*** |

*ρ*: Spearman´s correlation coefficient *rho* with $|\rho| \geq 0.1$ = small, $|\rho| \geq 0.3$ = medium and $|\rho| > 0.5$ = large effects. Significant *p*-values given in bold, italic.

**Table S6** Partial correlation of post-psilocybin application resting state oscillatory activity with alcohol consumption.

| Parameter | Alcohol consumption | ρ | p |
|---|---|---|---|
| deltaPower_dB | BL_Alc5 | -0.344 | 0.364 |
| | BL_Alc10 | 0.123 | 0.753 |
| | BL_Alc20 | -0.230 | 0.552 |
| | BL_TotalAlc | -0.462 | 0.210 |
| | ADE_Alc5 | -0.355 | 0.349 |
| | ADE_Alc10 | -0.614 | 0.079 |
| | ADE_Alc20 | 0.125 | 0.749 |
| | ADE_TotalAlc | -0.679 | ***0.044*** |
| | Relapse_Alc5 | -0.062 | 0.874 |
| | Relapse_Alc10 | -0.441 | 0.235 |
| | Relapse_Alc20 | 0.120 | 0.758 |
| | Relapse_TotalAlc | -0.273 | 0.477 |
| thetaPower_dB | BL_Alc5 | -0.146 | 0.707 |
| | BL_Alc10 | -0.368 | 0.330 |
| | BL_Alc20 | 0.432 | 0.245 |
| | BL_TotalAlc | -0.213 | 0.582 |
| | ADE_Alc5 | -0.498 | 0.172 |
| | ADE_Alc10 | -0.094 | 0.809 |
| | ADE_Alc20 | 0.099 | 0.800 |
| | ADE_TotalAlc | -0.576 | 0.104 |
| | Relapse_Alc5 | -0.432 | 0.245 |
| | Relapse_Alc10 | 0.445 | 0.230 |
| | Relapse_Alc20 | -0.353 | 0.351 |
| | Relapse_TotalAlc | -0.378 | 0.316 |
| alphaPower_dB | BL_Alc5 | -0.075 | 0.849 |
| | BL_Alc10 | -0.162 | 0.677 |
| | BL_Alc20 | 0.325 | 0.394 |
| | BL_TotalAlc | -0.075 | 0.848 |
| | ADE_Alc5 | -0.454 | 0.220 |
| | ADE_Alc10 | -0.120 | 0.759 |
| | ADE_Alc20 | -0.069 | 0.861 |
| | ADE_TotalAlc | -0.608 | 0.082 |
| | Relapse_Alc5 | -0.436 | 0.240 |
| | Relapse_Alc10 | 0.374 | 0.321 |
| | Relapse_Alc20 | -0.407 | 0.276 |
| | Relapse_TotalAlc | -0.624 | 0.073 |
| betaPower_dB | BL_Alc5 | -0.060 | 0.878 |
| | BL_Alc10 | -0.048 | 0.902 |
| | BL_Alc20 | 0.322 | 0.397 |
| | BL_TotalAlc | 0.020 | 0.959 |
| | ADE_Alc5 | -0.445 | 0.230 |
| | ADE_Alc10 | -0.143 | 0.713 |
| | ADE_Alc20 | 0.017 | 0.965 |
| | ADE_TotalAlc | -0.510 | 0.160 |
| | Relapse_Alc5 | -0.435 | 0.242 |
| | Relapse_Alc10 | 0.395 | 0.293 |
| | Relapse_Alc20 | -0.278 | 0.468 |
| | Relapse_TotalAlc | -0.608 | 0.082 |
| gammaPower_dB | BL_Alc5 | -0.144 | 0.712 |
| | BL_Alc10 | -0.025 | 0.949 |
| | BL_Alc20 | 0.242 | 0.531 |
| | BL_TotalAlc | -0.028 | 0.944 |
| | ADE_Alc5 | -0.494 | 0.177 |
| | ADE_Alc10 | -0.327 | 0.391 |
| | ADE_Alc20 | 0.087 | 0.823 |
| | ADE_TotalAlc | -0.565 | 0.113 |
| | Relapse_Alc5 | -0.306 | 0.423 |
| | Relapse_Alc10 | 0.203 | 0.600 |
| | Relapse_Alc20 | -0.232 | 0.549 |
| | Relapse_TotalAlc | -0.521 | 0.150 |

*ρ*: Spearman´s correlation coefficient *rho* with |*ρ*| ≥ 0.1 = small, |*ρ*| ≥ 0.3 = medium and |*ρ*| > 0.5 = large effects. Significant *p*-values given in bold, italic.

**Table S7** Impact of psilocybin on event-related neural activity compared to non-treated conditions in the ADE rat model.

| Parameter | df | F | p | $\eta_p^2$ |
|---|---|---|---|---|
| LatP1 | 1, 63 | 0.254 | 0.616 | 0.004 |
| LatN1 | 1, 63 | 1.199 | 0.278 | 0.019 |
| LatP2 | 1, 63 | 0 | 0.997 | 0.000 |
| LatN2 | 1, 63 | 10.937 | ***0.002*** | 0.148 |
| LatP3 | 1, 63 | 12.695 | ***0.001*** | 0.168 |
| AmplP1N1 | 1, 63 | 33.973 | ***0.000*** | 0.350 |
| AmplN1P2 | 1, 63 | 28.777 | ***0.000*** | 0.314 |
| AmplP2N2 | 1, 63 | 1.668 | 0.201 | 0.026 |
| AmplN2P3 | 1, 63 | 23.676 | ***0.000*** | 0.273 |
| deltaPower_dB | 1, 63 | 30.308 | ***0.000*** | 0.325 |
| thetaPower_dB | 1, 63 | 38.971 | ***0.000*** | 0.382 |
| alphaPower_dB | 1, 63 | 40.054 | ***0.000*** | 0.389 |
| betaPower_dB | 1, 63 | 42.152 | ***0.000*** | 0.401 |
| gammaPower_dB | 1, 63 | 28.403 | ***0.000*** | 0.311 |
| TotalPower_dB | 1, 63 | 35.477 | ***0.000*** | 0.360 |
| Time_deltaPower_max | 1, 63 | 1.144 | 0.289 | 0.018 |
| Freq_deltaPower_max | 1, 63 | 0.106 | 0.746 | 0.002 |
| Time_thetaPower_max | 1, 63 | 0.143 | 0.707 | 0.002 |
| Freq_thetaPower_max | 1, 63 | 9.944 | ***0.002*** | 0.136 |
| Time_alphaPower_max | 1, 63 | 0.015 | 0.903 | 0.000 |
| Freq_alphaPower_max | 1, 63 | 0.7 | 0.406 | 0.011 |
| Time_betaPower_max | 1, 63 | 2.523 | 0.117 | 0.039 |
| Freq_betaPower_max | 1, 63 | 30.249 | ***0.000*** | 0.324 |
| Time_gammaPower_max | 1, 63 | 1.259 | 0.266 | 0.020 |
| Freq_gammaPower_max | 1, 63 | 5.398 | ***0.023*** | 0.079 |
| Time_TotalPower_max | 1, 63 | 0.123 | 0.727 | 0.002 |
| Freq_TotalPower_max | 1, 63 | 3.607 | 0.062 | 0.054 |

ANOVA – main effect of treatment (no effect of channel location or treatment*channel interaction), df: degrees of freedom, $\eta_p^2$: effect size partial eta squared with $\eta_p^2 \geq 0.01$ = small, $\eta_p^2 \geq 0.06$ = medium and $\eta_p^2 > 0.14$ = large effects. Significant *p*-values given in bold, italic.

**Table S8** Impact of psilocybin on event-related neural activity in the ADE rat model compared to alcohol- and drug-naïve controls.

| **Parameter** | **df** | **F** | **p** | $\eta_p^2$ |
|---|---|---|---|---|
| LatP1 | 1, 149 | 9.361 | ***0.003*** | 0.059 |
| LatN1 | 1, 149 | 117.941 | ***0.000*** | 0.442 |
| LatP2 | 1, 149 | 23.524 | ***0.000*** | 0.136 |
| LatN2 | 1, 149 | 26.086 | ***0.000*** | 0.149 |
| LatP3 | 1, 149 | 13.765 | ***0.000*** | 0.085 |
| AmplP1N1 | 1, 149 | 11.888 | ***0.001*** | 0.074 |
| AmplN1P2 | 1, 149 | 10.371 | ***0.002*** | 0.065 |
| AmplP2N2 | 1, 149 | 14.011 | ***0.000*** | 0.086 |
| AmplN2P3 | 1, 149 | 8.507 | ***0.004*** | 0.054 |
| deltaPower_dB | 1, 149 | 1.986 | 0.161 | 0.013 |
| thetaPower_dB | 1, 149 | 2.256 | 0.135 | 0.015 |
| alphaPower_dB | 1, 149 | 2.559 | 0.112 | 0.017 |
| betaPower_dB | 1, 149 | 6.572 | ***0.011*** | 0.042 |
| gammaPower_dB | 1, 149 | 66.529 | ***0.000*** | 0.309 |
| TotalPower_dB | 1, 149 | 2.469 | 0.118 | 0.016 |
| Time_deltaPower_max | 1, 149 | 2.133 | 0.146 | 0.014 |
| Freq_deltaPower_max | 1, 149 | 5.601 | ***0.019*** | 0.036 |
| Time_thetaPower_max | 1, 149 | 3.126 | 0.079 | 0.021 |
| Freq_thetaPower_max | 1, 149 | 0.091 | 0.763 | 0.001 |
| Time_alphaPower_max | 1, 149 | 0.175 | 0.677 | 0.001 |
| Freq_alphaPower_max | 1, 149 | 0.539 | 0.464 | 0.004 |
| Time_betaPower_max | 1, 149 | 1.552 | 0.215 | 0.010 |
| Freq_betaPower_max | 1, 149 | 15.065 | ***0.000*** | 0.092 |
| Time_gammaPower_max | 1, 149 | 60.328 | ***0.000*** | 0.288 |
| Freq_gammaPower_max | 1, 149 | 7.763 | ***0.006*** | 0.050 |
| Time_Total_maxPower | 1, 149 | 0.505 | 0.479 | 0.003 |
| Freq_Total_maxPower | 1, 149 | 11.742 | ***0.001*** | 0.073 |

ANOVA – main effect of treatment (no effect of channel location or treatment*channel interaction), df: degrees of freedom, $\eta_p^2$: effect size partial eta squared with $\eta_p^2 \geq 0.01$ = small, $\eta_p^2 \geq 0.06$ = medium and $\eta_p^2 > 0.14$ = large effects. Significant *p*-values given in bold, italic.

**Table S9** Correlation of event-related neural activity following psilocybin application with non-treated conditions in the ADE rat model.

| **Parameter** | ***ρ*** | **p** |
|---|---|---|
| AmplP1N1 | 0.383 | 0.308 |
| AmplN1P2 | 0.200 | 0.606 |
| AmplP2N2 | -0.683 | ***0.042*** |
| AmplN2P3 | 0.150 | 0.700 |
| deltaPower_dB | 0.400 | 0.286 |
| thetaPower_dB | 0.500 | 0.170 |
| alphaPower_dB | 0.350 | 0.356 |
| betaPower_dB | 0.267 | 0.488 |
| gammaPower_dB | 0.550 | 0.125 |
| TotalPower_dB | 0.450 | 0.224 |

*ρ*: Spearman´s correlation coefficient *rho* with $|\rho| \geq 0.1$ = small, $|\rho| \geq 0.3$ = medium and $|\rho| > 0.5$ = large effects. Significant *p*-values given in bold, italic.

**Table S10** Partial correlation of event-related activity following psilocybin application with alcohol consumption in the ADE rat model.

| Parameter | Alcohol consumption | *ρ* | p |
|---|---|---|---|
| AmplP1N1 | BL_Alc5 | -0.406 | 0.318 |
| | BL_Alc10 | -0.306 | 0.461 |
| | BL_Alc20 | 0.229 | 0.585 |
| | BL_TotalAlc | -0.578 | 0.133 |
| | ADE_Alc5 | -0.649 | 0.082 |
| | ADE_Alc10 | -0.242 | 0.564 |
| | ADE_Alc20 | 0.454 | 0.258 |
| | ADE_TotalAlc | -0.382 | 0.351 |
| | Relapse_Alc5 | -0.391 | 0.339 |
| | Relapse_Alc10 | 0.168 | 0.691 |
| | Relapse_Alc20 | 0.232 | 0.580 |
| | Relapse_TotalAlc | 0.004 | 0.993 |
| AmplN1P2 | BL_Alc5 | -0.142 | 0.738 |
| | BL_Alc10 | -0.690 | 0.058 |
| | BL_Alc20 | 0.233 | 0.578 |
| | BL_TotalAlc | -0.887 | ***0.003*** |
| | ADE_Alc5 | -0.308 | 0.458 |
| | ADE_Alc10 | -0.674 | 0.067 |
| | ADE_Alc20 | 0.290 | 0.486 |
| | ADE_TotalAlc | -0.612 | 0.107 |
| | Relapse_Alc5 | -0.273 | 0.513 |
| | Relapse_Alc10 | 0.293 | 0.481 |
| | Relapse_Alc20 | 0.070 | 0.869 |
| | Relapse_TotalAlc | -0.078 | 0.853 |
| AmplP2N2 | BL_Alc5 | -0.099 | 0.816 |
| | BL_Alc10 | -0.091 | 0.831 |
| | BL_Alc20 | 0.616 | 0.104 |
| | BL_TotalAlc | 0.402 | 0.323 |
| | ADE_Alc5 | -0.005 | 0.991 |
| | ADE_Alc10 | 0.189 | 0.654 |
| | ADE_Alc20 | -0.327 | 0.429 |
| | ADE_TotalAlc | -0.220 | 0.601 |
| | Relapse_Alc5 | 0.255 | 0.543 |
| | Relapse_Alc10 | 0.183 | 0.664 |
| | Relapse_Alc20 | -0.749 | ***0.033*** |
| | Relapse_TotalAlc | -0.418 | 0.302 |
| AmplN2P3 | BL_Alc5 | -0.348 | 0.398 |
| | BL_Alc10 | -0.443 | 0.272 |
| | BL_Alc20 | 0.683 | 0.062 |
| | BL_TotalAlc | -0.186 | 0.659 |
| | ADE_Alc5 | -0.752 | ***0.031*** |
| | ADE_Alc10 | 0.128 | 0.762 |
| | ADE_Alc20 | 0.396 | 0.331 |
| | ADE_TotalAlc | -0.328 | 0.427 |
| | Relapse_Alc5 | -0.588 | 0.125 |
| | Relapse_Alc10 | 0.608 | 0.110 |
| | Relapse_Alc20 | -0.111 | 0.793 |
| | Relapse_TotalAlc | -0.160 | 0.705 |
| deltaPower_dB | BL_Alc5 | -0.479 | 0.230 |
| | BL_Alc10 | -0.579 | 0.133 |
| | BL_Alc20 | 0.429 | 0.288 |
| | BL_TotalAlc | -0.840 | ***0.009*** |
| | ADE_Alc5 | -0.574 | 0.137 |
| | ADE_Alc10 | -0.460 | 0.251 |
| | ADE_Alc20 | 0.575 | 0.136 |
| | ADE_TotalAlc | -0.430 | 0.287 |
| | Relapse_Alc5 | -0.201 | 0.632 |
| | Relapse_Alc10 | 0.218 | 0.604 |
| | Relapse_Alc20 | 0.182 | 0.666 |
| | Relapse_TotalAlc | 0.181 | 0.668 |

| Parameter | Alcohol consumption | *ρ* | p |
|---|---|---|---|
| thetaPower_dB | BL_Alc5 | -0.534 | 0.172 |
| | BL_Alc10 | -0.550 | 0.157 |
| | BL_Alc20 | 0.408 | 0.315 |
| | BL_TotalAlc | -0.799 | ***0.017*** |
| | ADE_Alc5 | -0.621 | 0.100 |
| | ADE_Alc10 | -0.406 | 0.318 |
| | ADE_Alc20 | 0.656 | 0.077 |
| | ADE_TotalAlc | -0.377 | 0.358 |
| | Relapse_Alc5 | -0.196 | 0.642 |
| | Relapse_Alc10 | 0.163 | 0.700 |
| | Relapse_Alc20 | 0.251 | 0.548 |
| | Relapse_TotalAlc | 0.291 | 0.485 |
| alphaPower_dB | BL_Alc5 | -0.439 | 0.276 |
| | BL_Alc10 | -0.558 | 0.151 |
| | BL_Alc20 | 0.416 | 0.305 |
| | BL_TotalAlc | -0.826 | ***0.012*** |
| | ADE_Alc5 | -0.606 | 0.111 |
| | ADE_Alc10 | -0.411 | 0.312 |
| | ADE_Alc20 | 0.610 | 0.108 |
| | ADE_TotalAlc | -0.398 | 0.329 |
| | Relapse_Alc5 | -0.288 | 0.489 |
| | Relapse_Alc10 | 0.284 | 0.495 |
| | Relapse_Alc20 | 0.251 | 0.549 |
| | Relapse_TotalAlc | 0.188 | 0.655 |
| betaPower_dB | BL_Alc5 | -0.462 | 0.249 |
| | BL_Alc10 | -0.378 | 0.356 |
| | BL_Alc20 | 0.381 | 0.352 |
| | BL_TotalAlc | -0.658 | 0.076 |
| | ADE_Alc5 | -0.731 | ***0.039*** |
| | ADE_Alc10 | -0.276 | 0.508 |
| | ADE_Alc20 | 0.637 | 0.090 |
| | ADE_TotalAlc | -0.350 | 0.396 |
| | Relapse_Alc5 | -0.424 | 0.295 |
| | Relapse_Alc10 | 0.294 | 0.479 |
| | Relapse_Alc20 | 0.311 | 0.453 |
| | Relapse_TotalAlc | 0.102 | 0.810 |
| gammaPower_dB | BL_Alc5 | -0.477 | 0.232 |
| | BL_Alc10 | 0.080 | 0.850 |
| | BL_Alc20 | 0.338 | 0.413 |
| | BL_TotalAlc | -0.222 | 0.598 |
| | ADE_Alc5 | -0.835 | ***0.010*** |
| | ADE_Alc10 | 0.024 | 0.956 |
| | ADE_Alc20 | 0.606 | 0.111 |
| | ADE_TotalAlc | -0.151 | 0.722 |
| | Relapse_Alc5 | -0.487 | 0.221 |
| | Relapse_Alc10 | 0.256 | 0.541 |
| | Relapse_Alc20 | 0.302 | 0.468 |
| | Relapse_TotalAlc | -0.031 | 0.942 |
| TotalPower_dB | BL_Alc5 | -0.435 | 0.281 |
| | BL_Alc10 | -0.546 | 0.161 |
| | BL_Alc20 | 0.350 | 0.396 |
| | BL_TotalAlc | -0.856 | ***0.007*** |
| | ADE_Alc5 | -0.596 | 0.119 |
| | ADE_Alc10 | -0.446 | 0.268 |
| | ADE_Alc20 | 0.614 | 0.105 |
| | ADE_TotalAlc | -0.402 | 0.323 |
| | Relapse_Alc5 | -0.277 | 0.506 |
| | Relapse_Alc10 | 0.200 | 0.634 |
| | Relapse_Alc20 | 0.293 | 0.481 |
| | Relapse_TotalAlc | 0.220 | 0.601 |

$\rho$: Spearman´s correlation coefficient *rho* with $|\rho| \geq 0.1$ = small, $|\rho| \geq 0.3$ = medium and $|\rho| > 0.5$ = large effects. Significant *p*-values given in bold, italic.

**Table S11** Impact of LY379268 on resting state oscillator activity in the ADE rat model.

| Parameter | df | F | p | $\eta_p^2$ |
|---|---|---|---|---|
| deltaPower_dB | 1, 76 | 28.549 | ***0.000*** | 0.273 |
| thetaPower_dB | 1, 76 | 20.18 | ***0.000*** | 0.210 |
| alphaPower_dB | 1, 76 | 30.67 | ***0.000*** | 0.288 |
| betaPower_dB | 1, 76 | 36.607 | ***0.000*** | 0.325 |
| gammaPower_dB | 1, 76 | 36.903 | ***0.000*** | 0.327 |

ANOVA – main effect of treatment (no effect of channel location or treatment*channel interaction), df: degrees of freedom, $\eta_p^2$: effect size partial eta squared with $\eta_p^2 \geq 0.01$ = small, $\eta_p^2 \geq 0.06$ = medium and $\eta_p^2 > 0.14$ = large effects. Significant *p*-values given in bold, italic.

**Table S12** Correlation of resting state oscillatory activity after vs. before LY379268 application in the ADE rat model.

| Parameter | $\rho$ | p |
|---|---|---|
| deltaPower_dB | 0.394 | 0.260 |
| thetaPower_dB | 0.188 | 0.603 |
| alphaPower_dB | 0.152 | 0.676 |
| betaPower_dB | 0.552 | 0.098 |
| gammaPower_dB | 0.661 | ***0.038*** |

$\rho$: Spearman´s correlation coefficient *rho* with $|\rho| \geq 0.1$ = small, $|\rho| \geq 0.3$ = medium and $|\rho| > 0.5$ = large effects. Significant *p*-values given in bold, italic.

**Table S13** Partial correlation of post-LY379268 application resting state oscillatory activity with alcohol consumption.

| Parameter | Alcohol consumption | *ρ* | p |
|---|---|---|---|
| deltaPower_dB | BL_Alc5 | -0.365 | 0.334 |
| | BL_Alc10 | 0.420 | 0.261 |
| | BL_Alc20 | -0.351 | 0.354 |
| | BL_TotalAlc | -0.364 | 0.336 |
| | ADE_Alc5 | -0.146 | 0.707 |
| | ADE_Alc10 | -0.404 | 0.280 |
| | ADE_Alc20 | 0.050 | 0.899 |
| | ADE_TotalAlc | -0.271 | 0.481 |
| | Relapse_Alc5 | 0.430 | 0.249 |
| | Relapse_Alc10 | -0.776 | ***0.014*** |
| | Relapse_Alc20 | 0.210 | 0.587 |
| | Relapse_TotalAlc | -0.137 | 0.726 |
| thetaPower_dB | BL_Alc5 | -0.353 | 0.352 |
| | BL_Alc10 | -0.130 | 0.739 |
| | BL_Alc20 | 0.487 | 0.184 |
| | BL_TotalAlc | -0.249 | 0.518 |
| | ADE_Alc5 | -0.320 | 0.401 |
| | ADE_Alc10 | -0.086 | 0.826 |
| | ADE_Alc20 | 0.090 | 0.818 |
| | ADE_TotalAlc | -0.324 | 0.396 |
| | Relapse_Alc5 | 0.038 | 0.922 |
| | Relapse_Alc10 | -0.017 | 0.965 |
| | Relapse_Alc20 | -0.221 | 0.568 |
| | Relapse_TotalAlc | -0.171 | 0.661 |
| alphaPower_dB | BL_Alc5 | -0.601 | 0.087 |
| | BL_Alc10 | 0.080 | 0.837 |
| | BL_Alc20 | 0.406 | 0.278 |
| | BL_TotalAlc | -0.369 | 0.329 |
| | ADE_Alc5 | -0.577 | 0.104 |
| | ADE_Alc10 | -0.100 | 0.798 |
| | ADE_Alc20 | 0.313 | 0.413 |
| | ADE_TotalAlc | -0.353 | 0.352 |
| | Relapse_Alc5 | -0.021 | 0.957 |
| | Relapse_Alc10 | -0.225 | 0.561 |
| | Relapse_Alc20 | -0.048 | 0.902 |
| | Relapse_TotalAlc | -0.069 | 0.859 |
| betaPower_dB | BL_Alc5 | -0.391 | 0.298 |
| | BL_Alc10 | 0.311 | 0.416 |
| | BL_Alc20 | 0.056 | 0.885 |
| | BL_TotalAlc | -0.152 | 0.697 |
| | ADE_Alc5 | -0.370 | 0.327 |
| | ADE_Alc10 | 0.075 | 0.848 |
| | ADE_Alc20 | -0.025 | 0.948 |
| | ADE_TotalAlc | -0.200 | 0.607 |
| | Relapse_Alc5 | 0.081 | 0.837 |
| | Relapse_Alc10 | -0.329 | 0.388 |
| | Relapse_Alc20 | -0.085 | 0.827 |
| | Relapse_TotalAlc | -0.147 | 0.705 |
| gammaPower_dB | BL_Alc5 | -0.434 | 0.243 |
| | BL_Alc10 | 0.239 | 0.535 |
| | BL_Alc20 | -0.199 | 0.608 |
| | BL_TotalAlc | -0.335 | 0.379 |
| | ADE_Alc5 | -0.214 | 0.580 |
| | ADE_Alc10 | 0.024 | 0.952 |
| | ADE_Alc20 | 0.013 | 0.973 |
| | ADE_TotalAlc | -0.035 | 0.929 |
| | Relapse_Alc5 | 0.299 | 0.434 |
| | Relapse_Alc10 | -0.571 | 0.108 |
| | Relapse_Alc20 | 0.116 | 0.767 |
| | Relapse_TotalAlc | 0.181 | 0.641 |

*ρ*: Spearman´s correlation coefficient *rho* with $|\rho| \geq 0.1$ = small, $|\rho| \geq 0.3$ = medium and $|\rho| > 0.5$ = large effects. Significant *p*-values given in bold, italic.

**Table S14** Impact of L379268 on event-related neural activity compared to non-treated conditions in the ADE rat model.

| **Parameter** | **df** | **F** | **p** | $\eta_p^2$ |
|---|---|---|---|---|
| LatP1 | 1, 62 | 1.195 | 0.279 | 0.019 |
| LatN1 | 1, 62 | 5.807 | ***0.019*** | 0.086 |
| LatP2 | 1, 62 | 0.038 | 0.845 | 0.001 |
| LatN2 | 1, 62 | 10.89 | ***0.002*** | 0.149 |
| LatP3 | 1, 62 | 11.942 | ***0.001*** | 0.162 |
| AmplP1N1 | 1, 62 | 17.064 | ***0.000*** | 0.216 |
| AmplN1P2 | 1, 62 | 7.227 | ***0.009*** | 0.104 |
| AmplP2N2 | 1, 62 | 38.304 | ***0.000*** | 0.382 |
| AmplN2P3 | 1, 62 | 2.46 | 0.122 | 0.038 |
| deltaPower_dB | 1, 62 | 6.224 | ***0.015*** | 0.091 |
| thetaPower_dB | 1, 62 | 8.814 | ***0.004*** | 0.124 |
| alphaPower_dB | 1, 62 | 10.361 | ***0.002*** | 0.143 |
| betaPower_dB | 1, 62 | 6.999 | ***0.010*** | 0.101 |
| gammaPower_dB | 1, 62 | 0.101 | 0.752 | 0.002 |
| TotalPower_dB | 1, 62 | 8.576 | ***0.005*** | 0.122 |
| Time_deltaPower_max | 1, 62 | 8.128 | ***0.006*** | 0.116 |
| Freq_deltaPower_max | 1, 62 | 0.306 | 0.582 | 0.005 |
| Time_thetaPower_max | 1, 62 | 5.768 | ***0.019*** | 0.085 |
| Freq_thetaPower_max | 1, 62 | 6.94 | ***0.011*** | 0.101 |
| Time_alphaPower_max | 1, 62 | 3.669 | 0.060 | 0.056 |
| Freq_alphaPower_max | 1, 62 | 0.403 | 0.528 | 0.006 |
| Time_betaPower_max | 1, 62 | 0.438 | 0.511 | 0.007 |
| Freq_betaPower_max | 1, 62 | 16.567 | ***0.000*** | 0.211 |
| Time_gammaPower_max | 1, 62 | 0.67 | 0.416 | 0.011 |
| Freq_gammaPower_max | 1, 62 | 12.623 | ***0.001*** | 0.169 |
| Time_TotalPower_max | 1, 62 | 2.116 | 0.151 | 0.033 |
| Freq_TotalPower_max | 1, 62 | 3.09 | 0.084 | 0.047 |

ANOVA – main effect of treatment (no effect of channel location or treatment*channel interaction), df: degrees of freedom, $\eta_p^2$: effect size partial eta squared with $\eta_p^2 \geq 0.01$ = small, $\eta_p^2 \geq 0.06$ = medium and $\eta_p^2 > 0.14$ = large effects. Significant *p*-values given in bold, italic.

**Table S15** Impact of L379268 on event-related neural activity in the ADE rat model compared to alcohol- and drug-naïve controls.

| Parameter | df | F | p | $\eta_p^2$ |
|---|---|---|---|---|
| LatP1 | 1, 148 | 9.576 | ***0.002*** | 0.061 |
| LatN1 | 1, 148 | 17.313 | ***0.000*** | 0.105 |
| LatP2 | 1, 148 | 21.535 | ***0.000*** | 0.127 |
| LatN2 | 1, 148 | 23.620 | ***0.000*** | 0.138 |
| LatP3 | 1, 148 | 17.290 | ***0.000*** | 0.105 |
| AmplP1N1 | 1, 148 | 0.222 | 0.638 | 0.002 |
| AmplN1P2 | 1, 148 | 0.389 | 0.534 | 0.003 |
| AmplP2N2 | 1, 148 | 6.408 | ***0.012*** | 0.041 |
| AmplN2P3 | 1, 148 | 1.261 | 0.263 | 0.008 |
| deltaPower_dB | 1, 148 | 2.081 | 0.151 | 0.014 |
| thetaPower_dB | 1, 148 | 2.394 | 0.124 | 0.016 |
| alphaPower_dB | 1, 148 | 1.942 | 0.166 | 0.013 |
| betaPower_dB | 1, 148 | 1.387 | 0.241 | 0.009 |
| gammaPower_dB | 1, 148 | 9.336 | ***0.003*** | 0.059 |
| TotalPower_dB | 1, 148 | 2.246 | 0.136 | 0.015 |
| Time_deltaPower_max | 1, 148 | 0.780 | 0.379 | 0.005 |
| Freq_deltaPower_max | 1, 148 | 2.293 | 0.132 | 0.015 |
| Time_thetaPower_max | 1, 148 | 0.739 | 0.391 | 0.005 |
| Freq_thetaPower_max | 1, 148 | 0.001 | 0.980 | 0.000 |
| Time_alphaPower_max | 1, 148 | 1.382 | 0.242 | 0.009 |
| Freq_alphaPower_max | 1, 148 | 0.008 | 0.929 | 0.000 |
| Time_betaPower_max | 1, 148 | 3.546 | 0.062 | 0.023 |
| Freq_betaPower_max | 1, 148 | 13.930 | ***0.000*** | 0.086 |
| Time_gammaPower_max | 1, 148 | 53.938 | ***0.000*** | 0.267 |
| Freq_gammaPower_max | 1, 148 | 0.073 | 0.788 | 0.000 |
| Time_Total_maxPower | 1, 148 | 0.805 | 0.371 | 0.005 |
| Freq_Total_maxPower | 1, 148 | 4.785 | ***0.030*** | 0.031 |

ANOVA – main effect of treatment (no effect of channel location or treatment*channel interaction), df: degrees of freedom, $\eta_p^2$: effect size partial eta squared with $\eta_p^2 \geq 0.01$ = small, $\eta_p^2 \geq 0.06$ = medium and $\eta_p^2 > 0.14$ = large effects. Significant *p*-values given in bold, italic.

**Table S16** Correlation of event-related neural activity following LY379268 application with non-treated conditions in the ADE rat model.

| Parameter | ***ρ*** | **p** |
|---|---|---|
| AmplP1N1 | 0.383 | 0.308 |
| AmplN1P2 | 0.300 | 0.433 |
| AmplP2N2 | -0.067 | 0.865 |
| AmplP1N1 | 0.017 | 0.966 |
| deltaPower_dB | 0.250 | 0.516 |
| thetaPower_dB | 0.483 | 0.187 |
| alphaPower_dB | 0.250 | 0.516 |
| betaPower_dB | 0.267 | 0.488 |
| gammaPower_dB | 0.533 | 0.139 |
| TotalPower_dB | 0.333 | 0.381 |

$\rho$: Spearman´s correlation coefficient *rho* with $|\rho| \geq 0.1$ = small, $|\rho| \geq 0.3$ = medium and $|\rho| > 0.5$ = large effects. Significant *p*-values given in bold, italic.

**Table S17** Partial correlation of event-related activity following L379268 application with alcohol consumption in the ADE rat model.

| Parameter | Alcohol consumption | *ρ* | p |
|---|---|---|---|
| AmplP1N1 | BL_Alc5 | -0.166 | 0.694 |
| | BL_Alc10 | -0.459 | 0.252 |
| | BL_Alc20 | 0.144 | 0.734 |
| | BL_TotalAlc | -0.614 | 0.105 |
| | ADE_Alc5 | -0.156 | 0.712 |
| | ADE_Alc10 | -0.314 | 0.449 |
| | ADE_Alc20 | 0.110 | 0.795 |
| | ADE_TotalAlc | -0.326 | 0.430 |
| | Relapse_Alc5 | -0.016 | 0.970 |
| | Relapse_Alc10 | -0.034 | 0.937 |
| | Relapse_Alc20 | 0.085 | 0.842 |
| | Relapse_TotalAlc | 0.041 | 0.924 |
| AmplN1P2 | BL_Alc5 | -0.130 | 0.759 |
| | BL_Alc10 | -0.284 | 0.496 |
| | BL_Alc20 | 0.070 | 0.869 |
| | BL_TotalAlc | -0.480 | 0.228 |
| | ADE_Alc5 | -0.368 | 0.370 |
| | ADE_Alc10 | -0.017 | 0.968 |
| | ADE_Alc20 | 0.096 | 0.820 |
| | ADE_TotalAlc | -0.297 | 0.475 |
| | Relapse_Alc5 | -0.253 | 0.545 |
| | Relapse_Alc10 | 0.168 | 0.691 |
| | Relapse_Alc20 | 0.063 | 0.882 |
| | Relapse_TotalAlc | 0.026 | 0.952 |
| AmplP2N2 | BL_Alc5 | -0.049 | 0.908 |
| | BL_Alc10 | -0.220 | 0.600 |
| | BL_Alc20 | 0.367 | 0.370 |
| | BL_TotalAlc | 0.045 | 0.916 |
| | ADE_Alc5 | 0.445 | 0.269 |
| | ADE_Alc10 | -0.205 | 0.626 |
| | ADE_Alc20 | -0.567 | 0.143 |
| | ADE_TotalAlc | -0.380 | 0.353 |
| | Relapse_Alc5 | 0.645 | 0.084 |
| | Relapse_Alc10 | -0.294 | 0.480 |
| | Relapse_Alc20 | -0.880 | ***0.004*** |
| | Relapse_TotalAlc | -0.345 | 0.403 |
| AmplN2P3 | BL_Alc5 | -0.445 | 0.269 |
| | BL_Alc10 | -0.578 | 0.134 |
| | BL_Alc20 | 0.434 | 0.283 |
| | BL_TotalAlc | -0.822 | ***0.012*** |
| | ADE_Alc5 | -0.535 | 0.172 |
| | ADE_Alc10 | -0.558 | 0.150 |
| | ADE_Alc20 | 0.379 | 0.354 |
| | ADE_TotalAlc | -0.661 | 0.075 |
| | Relapse_Alc5 | -0.232 | 0.580 |
| | Relapse_Alc10 | 0.084 | 0.843 |
| | Relapse_Alc20 | -0.085 | 0.841 |
| | Relapse_TotalAlc | -0.252 | 0.547 |
| deltaPower_db | BL_Alc5 | -0.310 | 0.455 |
| | BL_Alc10 | -0.215 | 0.609 |
| | BL_Alc20 | 0.186 | 0.659 |
| | BL_TotalAlc | -0.497 | 0.210 |
| | ADE_Alc5 | -0.401 | 0.325 |
| | ADE_Alc10 | -0.143 | 0.736 |
| | ADE_Alc20 | 0.168 | 0.691 |
| | ADE_TotalAlc | -0.328 | 0.428 |
| | Relapse_Alc5 | -0.069 | 0.871 |
| | Relapse_Alc10 | -0.044 | 0.918 |
| | Relapse_Alc20 | 0.017 | 0.968 |
| | Relapse_TotalAlc | -0.023 | 0.956 |

| Parameter | Alcohol consumption | *ρ* | p |
|---|---|---|---|
| thetaPower_dB | BL_Alc5 | -0.250 | 0.550 |
| | BL_Alc10 | -0.164 | 0.698 |
| | BL_Alc20 | 0.047 | 0.912 |
| | BL_TotalAlc | -0.429 | 0.288 |
| | ADE_Alc5 | -0.406 | 0.318 |
| | ADE_Alc10 | -0.017 | 0.968 |
| | ADE_Alc20 | 0.132 | 0.755 |
| | ADE_TotalAlc | -0.279 | 0.504 |
| | Relapse_Alc5 | -0.118 | 0.781 |
| | Relapse_Alc10 | -0.059 | 0.890 |
| | Relapse_Alc20 | 0.055 | 0.896 |
| | Relapse_TotalAlc | 0.044 | 0.918 |
| alphaPower_dB | BL_Alc5 | -0.070 | 0.869 |
| | BL_Alc10 | -0.280 | 0.502 |
| | BL_Alc20 | 0.014 | 0.973 |
| | BL_TotalAlc | -0.496 | 0.211 |
| | ADE_Alc5 | -0.285 | 0.493 |
| | ADE_Alc10 | -0.057 | 0.893 |
| | ADE_Alc20 | 0.061 | 0.887 |
| | ADE_TotalAlc | -0.280 | 0.502 |
| | Relapse_Alc5 | -0.172 | 0.683 |
| | Relapse_Alc10 | 0.114 | 0.788 |
| | Relapse_Alc20 | 0.083 | 0.845 |
| | Relapse_TotalAlc | 0.014 | 0.973 |
| betaPower_dB | BL_Alc5 | 0.049 | 0.907 |
| | BL_Alc10 | -0.500 | 0.207 |
| | BL_Alc20 | 0.005 | 0.990 |
| | BL_TotalAlc | -0.639 | 0.088 |
| | ADE_Alc5 | -0.178 | 0.674 |
| | ADE_Alc10 | -0.189 | 0.654 |
| | ADE_Alc20 | 0.077 | 0.856 |
| | ADE_TotalAlc | -0.315 | 0.447 |
| | Relapse_Alc5 | -0.198 | 0.638 |
| | Relapse_Alc10 | 0.241 | 0.565 |
| | Relapse_Alc20 | 0.147 | 0.728 |
| | Relapse_TotalAlc | 0.083 | 0.845 |
| gammaPower_dB | BL_Alc5 | -0.176 | 0.677 |
| | BL_Alc10 | -0.520 | 0.187 |
| | BL_Alc20 | 0.146 | 0.730 |
| | BL_TotalAlc | -0.667 | 0.071 |
| | ADE_Alc5 | -0.122 | 0.773 |
| | ADE_Alc10 | -0.405 | 0.320 |
| | ADE_Alc20 | 0.122 | 0.774 |
| | ADE_TotalAlc | -0.354 | 0.390 |
| | Relapse_Alc5 | 0.035 | 0.935 |
| | Relapse_Alc10 | -0.100 | 0.814 |
| | Relapse_Alc20 | 0.080 | 0.850 |
| | Relapse_TotalAlc | 0.029 | 0.946 |
| TotalPower_dB | BL_Alc5 | -0.059 | 0.889 |
| | BL_Alc10 | -0.260 | 0.534 |
| | BL_Alc20 | -0.069 | 0.871 |
| | BL_TotalAlc | -0.510 | 0.196 |
| | ADE_Alc5 | -0.263 | 0.529 |
| | ADE_Alc10 | -0.072 | 0.865 |
| | ADE_Alc20 | 0.047 | 0.912 |
| | ADE_TotalAlc | -0.277 | 0.507 |
| | Relapse_Alc5 | -0.156 | 0.712 |
| | Relapse_Alc10 | 0.039 | 0.928 |
| | Relapse_Alc20 | 0.108 | 0.799 |
| | Relapse_TotalAlc | 0.039 | 0.928 |

*ρ*: Spearman´s correlation coefficient *rho* with $|\rho| \geq 0.1$ = small, $|\rho| \geq 0.3$ = medium and $|\rho| > 0.5$ = large effects. Significant *p*-values given in bold, italic.
